# Supplementary material for: Tuning polymer-backbone coplanarity and conformational order to achieve high-performance printed all-polymer solar cells
Source: Nat Commun. 2024 Mar 9;15:2170. doi: 10.1038/s41467-024-46493-4 (PMC10924936; doi:10.1038/s41467-024-46493-4)
Supplement: Supplementary file 1 — Supplementary Information [file 41467_2024_46493_MOESM1_ESM.pdf]

Supplementary Information
For

Tuning Polymer-Backbone Coplanarity and Conformational Order to Achieve High-Performance Printed All-Polymer Solar Cells

Yilei Wu[1], Yue Yuan[2], Diego Sorbelli[3], Christina Cheng[4], Lukas Michalek[1], Hao-Wen Cheng[1], Vishal Jindal[5], Song Zhang[1], Garrett LeCroy[4], Enrique D. Gomez[6], Scott T. Milner[5], Alberto Salleo[4], Giulia Galli[3], John B. Asbury[2], Michael F. Toney[7] and Zhenan Bao[1]

[1]Department of Chemical Engineering, Stanford University, Stanford, CA 94305-4125, USA.

[2]Department of Chemistry, The Pennsylvania State University, University Park, Pennsylvania 16802, USA.

[3]Pritzker School of Molecular Engineering, University of Chicago, 5747 South Ellis Avenue, Chicago, Illinois 60637, USA.

[4]Department of Materials Science and Engineering, Stanford University, Stanford, CA 94305, USA.

[5]Department of Chemical Engineering, The Pennsylvania State University, University Park, Pennsylvania 16802, USA.

[6]Department of Chemical Engineering and Department of Materials Science and Engineering, The Pennsylvania State University, University Park, Pennsylvania 16802, USA.

[7]Department of Chemical and Biological Engineering, University of Colorado Boulder, Boulder, Colorado 80309, USA.

Email: zbao@stanford.edu

Table of Contents

Supplementary Methods
Supplementary Figures 1-29
Supplementary Tables 1-5
Supplementary References

Supplementary Methods

All solvents were purchased from commercial suppliers and used without further purification unless stated otherwise. Thin layer chromatography (TLC) was performed on silica gel 60 F254 (E. Merck). Column chromatography was carried out on silica gel 60F (Merck 9385, 0.040–0.063 mm). Nuclear magnetic resonance (NMR) spectra were recorded on a Bruker Avance 500 spectrometer, with working frequencies of 500 MHz (^{1}H) and at 125 MHz (^{13}C), respectively. Chemical shifts are reported in ppm relative to the signals corresponding to the residual non-deuterated solvents ($\delta CDCl_3$ = 7.26 ppm, δCD_2Cl_2 = 5.32 ppm). MALDI measurements are conducted on a Bruker Microflex MALDI-TOF spectrometer. Polymer samples are prepared by dissolving the polymers and the MALDI matrix (trans-2-[3-(4-tert-Butylphenyl)-2-methyl-2-propenylidene]malononitrile (DCTB)) in chlorobenzene and spotted the solution on the MALDI plate. Thermogravimetric analysis (TGA) and differential scanning calorimetry (DSC) were carried out under N_2 flow on a TA Instrument Q100 and Mettler Toledon AG-TGA/SDTA851e model, respectively. Electrochemical experiments were performed with a CH Instruments 600E potentiostat. Photoelectron spectroscopy in air (PESA) measurements were taken on a Riken AC-2 photoelectron spectrometer with a power setting of 5 nW and a power number of 0.5. UV/Vis/NIR absorption spectra were recorded using a Cary 6000i spectrophotometer. PL spectra were acquired using HORIBA Fluorolog3 spectrofluorometer equipped with a picosecond TCSPC module.

An ultrafast transient absorption system with a tunable pump and white-light probe was used to measure the differential absorption through the sample. The laser system consists of a regeneratively amplified Ti:sapphire oscillator (Coherent Libra), which delivers 4 mJ pulse energies centred at 800 nm with a 1 kHz repetition rate. The pulse duration of the amplified pulse is approximately 50 fs. The laser output was split by an optical wedge to produce the pump and probe beams and the pump beam wavelength was tuned by an optical parametric amplifier (Coherent OPerA). The pump beam was focused onto the sample by spherical lens at near-normal incidence (spot size FWHM ~ 300 µm). The probe beam was focused onto a sapphire plate to generate a white-light continuum probe, which was collected and refocused onto the sample by a spherical mirror (spot size FWHM ~ 150 µm). The transmitted white light was collected and analyzed with a commercial absorption spectrometer (Helios, Ultrafast Systems LLC). Pulse-to-pulse fluctuations of the white light continuum were accounted for by a simultaneous reference

measurement of the continuum. The pump wavelength was maintained at 800 nm with a pulse power of 100 nJ (or approximately 80 μJ/cm^2). Pump and probe beam were linearly cross-polarized and any scattered pump-light into the detection path was filtered by a linear polarizer. The time delay was adjusted by delaying the pump pulse with a linear translation stage (minimum step size 16 fs). The individual component kinetic traces were fit to biexponential decays via least squares fitting. Nanosecond mid-IR transient absorption experiments were performed using an inspIRe transient absorption spectrometer from Magnitude Instruments (State College, PA). The instrument utilized the second harmonic of a nanosecond Nd:YAG laser (532 nm) to excite the samples. An infrared glowbar was used to produce the infrared probe light. Infrared probe radiation was focused on the sample, overlapped with the 532 nm pump pulse, and dispersed into a monochromator. A liquid N2 cooled mercury cadmium telluride (MCT) photovoltaic detector was used to collect the transient absorption data.

General Computational Settings: All DFT calculations have been carried out with the ORCA quantum chemistry program package (5.0.3 version)[1]. All geometry optimizations of monomer, dimer and trimer PY-2T and PY-BTz structures have been carried out *in vacuo* using the B3LYP.[2,3] exchange-correlation functional, the double-zeta quality def2-SVP basis set[4] and the D3 atom-pairwise dispersion correction with the Becke-Johnson damping scheme (D3BJ).[5,6] In all cases, structures have been simplified by replacing the alkyl side chains with methyl groups.

All properties (i.e. absolute energies, molecular electrostatic potential and atomic charges) have been evaluated by carrying out a single point calculation on optimized structures at the same level of theory but with a triple-zeta quality def2-TZVPP basis set[7]. Relaxed potential energy surface scans have been carried out with the same protocol (i.e., scanning at the def2-SVP level and refining the relative energies with the larger def2-TZVPP basis set). Atomic charges on the bithiophene/bithiazole units of PY-2T/PY-BTz monomers have been computed from the molecular electrostatic potential by using the Charges from Electrostatic Potentials using a Grid-based (CHELPG) method[8] as implemented in ORCA.

Morphology Characterizations: Ex-situ GIWAXS images were collected at beamline 11-3 of the Stanford Synchrotron Radiation Lightsource (SSRL) using 2D area detector in a helium chamber to minimize air-scattering background. The sample-to-detector distance was set to 320 mm and the incidence angle was 0.14°. The X-ray wavelength was 0.9758 Å (12.735 keV). The

samples were spin-coated on bare Si wafers with a thin layer of native oxide following the same recipe as for the device active layers. Three 180 s exposures were acquired per sample to achieve reasonable signal to noise for our analysis. The image was then converted into a reciprocal space map after being calibrated with a LaB6 reference sample.[9,10] The data were processed using python code developed within the group leveraging the pygix library.[11] We followed previously reported protocols to calculate the relative degree of crystallinity (rDoC).[12,13] The 2D-GIWAXS images of the respective samples were first multiplied by the geometrical correction factor $\sin(\chi)$ to account for crystallites at particular orientations of the polar angle χ.[13,14] The polar angle χ describes the relative orientation of crystallites with respect to the substrate normal. Here $\chi = 90^\circ$ and 0° are representing in-plane and out-of-plane orientations, respectively. Thus, considering the (100) peak $\chi = 90^\circ$ and 0° are corresponding to face-on and edge-on orientation of crystallites. The data were then background corrected and normalized for sample dimensions (scattering volume) as well as the incident X-ray intensity I_0 to account for differences in total scattering volume, largely driven by variations of film thickness, to facilitate a quantitative comparison between multiple samples. The crystal coherence length (CCL) was calculated based on Scherrer's equation CCL = $0.9 \cdot 2\pi \cdot (\Delta q)^{-1}$, where Δq is the full width at half-maximum (FWHM) of the respective polymer or small molecule peak.[15] The FWHM was extracted by fitting Gaussian or Voigt peaks to the data. CCL is a measure of the distance over which order is preserved in the crystalline material making the CCL value useful as proxy for the size of the crystalline domains. We use the CCL values to quantify the relative changes in crystallite dimensions, but the CCL values are not be interpreted as the actual physical size of crystallites. This is due to the fact that the peak FWHM does not adequately account for peak broadening stemming from para-crystalline disorder or instrumental broadening.[16]

RSoXS data were collected at Advanced Light Source (ALS) beamline 11.0.1.2 in transmission geometry under vacuum. For sample preparation, Si wafers were first spin-coated with poly(sodium 4-styrenesulfonate) (PSS) 10 wt% aqueous solution at 5000 rpm for 60 s. The substrates were then baked in air at 100 °C for 5 min to remove residual water. The desired BHJ ink was solution sheared or spin-coated on the PSS-coated Si wafer from 14 mg mL^{-1} chlorobenzene solution at 25 °C, then floated off in deionized water and picked up onto 100 nm Si_3N_4 membranes (Norcada Inc.). The film was then dried in air before being transferred into the vacuum chamber for RSoXS measurement. NEXAFS were taken from 270 to 350 eV and used to

calculate the energy dependent optical constants.[17] The contrast between two polymers was calculated from these and the scattering images were taken by a 2D CCD camera in vacuum at −45 °C (Princeton Instrument PI-MTE). Scattering data were collected at two sample-to-detector distances of 50 and 150 mm to give a combined q range of 0.001–0.070 $Å^{-1}$. Data analysis was also performed using the Nika package supported in the Igor Pro environment.[18]

The topographical images were determined using a Bruker Dimension Icon atomic force microscope in tapping mode. NSC15/Al-BS (MikroMasch, Tallinn, Estonia) AFM cantilever (typical resonant frequency of 325 kHz and force constant of 40 $N \cdot m^{-1}$) were employed for imaging the surface of the samples. The root-mean-square (RMS) surface roughness and phase differences were determined over a 2 × 2 μm^2 area which was measured with 512x512 pixels and a scan-rate of 0.8 Hz. AFM-QNM measurements were collected using a Bruker Icon Dimension instrument with NanoScope V electronics and a HQ:NSC19/Al BS probe (from MikroMasch with a nominal spring constant of 0.5 N/m, resonance frequency of 65 kHz, and a tip radius of 8 nm) which were calibrated. The calibration included the determination of the force constant (0.75 N/m) via thermal tuning, the deflection sensitivity (75.47 nm/V) against a sapphire reference sample and the tip radius (3.16nm) via tip qualification (NanoScope Analysis 3.0, Bruker) on a rough Ti reference sample. Nanomechanical maps were recorded in the Peak Force Quantitative Nanomechanics (PF-QNM) mode while scanning on a homogeneous area of the film surface. All nanomechanical images were recorded at a setpoint of ~ 800 pN with a Peak Force frequency of 2 kHz and amplitude of 150 nm. The scan resolution was set to 512x512 pixel with a scan-rate of 0.5 Hz for the 1um images and 256x256 pixels at 0.7 Hz for the 300nm images. The data was evaluated and depicted with Gwyddion SPM software.

Solar Cell Fabrication and Testing: Glass substrates with patterned ITO (20 mm × 20 mm) with a sheet resistance of 13 Ω /square were purchased from Xin Yan Technology Ltd. and used as received. After treating the substrate with UV–ozone for 20 min, ZnO was fabricated by spin-coating a diethylzinc precursor solution (15 wt % diethylzinc solution diluted 1:7 with tetrahydrofuran, both purchased from SigmaAldrich) was spin-coated onto the ITO surface at a speed of 5000 rpm for 30 s. The ZnO film was baked at 200 °C for 0.5 h in air to form a 30 nm thick layer. The polymers were dissolved in chlorobenzene and stirred at 80 °C for at least 8 h. The concentration was 15 mg mL^{-1} for donor and acceptors combined (1:1 ratio by weight). 1-chloronaphthalene (CN, 1% v/v) was added just before the film deposition as solvent additive, and

the thickness of the spin-coated active layer film was around 110 nm (1000 rpm for 1 min in air or N_2, followed by drying in N_2 for 2 min at 150 °C temperature). After film preparation, the samples were transferred to a vacuum evaporator for electrode deposition. A MoO_3 layer (7.5 nm) followed by an Ag layer (100 nm) were thermally deposited at a pressure of ca. 8×10^{-6} Torr. All devices were tested inside a nitrogen glove box under AM 1.5G illumination with an intensity of 100 mW cm^{-2} (Newport Solar Simulator 94021A) calibrated by a Newport certified silicon photodiode covered with a KG5 filter. The photodiode active area was 6.63 mm^2, which is comparable to the device effective area of 4.0 mm^2. The J–V curves were recorded with a Keithley 2400 semiconductor analyzer. External quantum efficiency (EQE) measurements were taken at short circuit using monochromated light from a tungsten lamp that was modulated by an optical chopper. The current from the devices was measured as a function of wavelength and compared to the current obtained from a photodiode with an NIST traceable calibration photocurrent action spectrum.

Single carrier devices were fabricated, and the dark current-voltage characteristics measured and analyzed in the space charge limited current (SCLC) regime. The structure of hole only devices was Glass/ITO/PEDOT:PSS/Active layer/MoO_3/Ag. For the electron only devices, the structure was Glass/ITO/ZnO/Active layer/LiF/Al. Mobilities were extracted by fitting the current density-voltage curves using the Mott-Gurney relationship. The reported mobility data are average values over the five devices of each sample. Highly sensitive FTPS-EQE measurements were performed following the process outlined by Vandewal et al., with a Nicolet iS50R FT-IR connected to a SRS Model SR570 low-noise current preamplifier.[19] For EL measurements, current was injected using a Keithley 2400 SMU, and output light was detected with the Ocean Insight QE Pro Spectrometer.

Single Crystal XRD Experimental: Single crystals of $C_{25.5}H_{33}N_{1.5}O_{1.5}S_{1.5}$ [Ph-BTz, CCDC 2269848] were grown by slow vapor diffusion of methanol in chlorobenzene solution. A suitable crystal was selected and mounted on a Bruker D8 Venture diffractometer. The crystal was kept at 100.00 K during data collection. Using Olex2[20], the structure was solved with the SHELXS[21] structure solution program using Direct Methods and refined with the SHELXL[22] refinement package using Least Squares minimization. **Crystal Data** for $C_{25.5}H_{33}N_{1.5}O_{1.5}S_{1.5}$ (M =432.62 g/mol): triclinic, space group P-1 (no. 2), a = 11.957(3) Å, b = 12.529(4) Å, c = 17.126(6) Å, α = 86.016(7)°, β = 84.547(7)°, γ = 63.378(9)°, V = 2282.3(12) Å^3, Z = 4, T = 100.00 K, μ(MoKα) = 0.209 mm^{-1}, $Dcalc$ = 1.259 g/cm^3, 164783 reflections measured (3.638° ≤ 2Θ ≤ 63.7°), 15644

unique (R_{int} = 0.1144, R_{sigma} = 0.0582) which were used in all calculations. The final R_1 was 0.0535 (I > 2σ(I)) and wR_2 was 0.1902 (all data).

General Procedures for the Synthesis of PY-BTz and PY-2T: Y6-OD-2Br (60 mg),[23] 2,2'-bis(trimethylstannyl)-5,5'-bithiazole (24.0 mg)[24] or 5,5'-bis(trimethylstannyl)-2,2'-bithiophene (16.9 mg),[25] $Pd_2(dba)_3$ (2 mol% vs. Y6-OD-2Br) and $P(o\text{-tolyl})_3$ (8 mol% vs. Y6-OD-2Br) were dissolved in dry chlorobenzene (3 mL) in a nitrogen glovebox. During the polymerization, the reaction system was protected from light. The reaction mixtures were stirred at 120 °C for 48 h., then they were cooled down to room temperature. The crude product was precipitated out in methanol, collected by filtering through Soxhlet thimble, and purified by extraction with hot methanol, acetone, and hexane under nitrogen for 12 h each. PY-BTz, M_n = 15.5 kDa, $Đ_M$ = 2.64; PY-2T, M_n = 15.7 kDa, $Đ_M$ = 2.62. The pure product fraction was collected and dried under vacuum before any characterization. MALDI-TOF, FTIR, and NMR spectra of **PY-BTz** and **PY-2T** are shown in Supplementary Figure 1, Supplementary Figure 2, and Supplementary Figure 4–9, respectively.

Procedures for the Synthesis of PBDB-TF*x*: different molar fractions of the fluorine-containing monomer units, 2,6-bis(trimethylstannyl)-4,8-bis(5-(2-ethylhexyl)-4-fluorothiophen-2-yl)benzo[1,2-b:4,5-b']dithiophene (0, 30.7, 61.3, 92.0, 122.7 mg, respectively) was used as the third monomer in the copolymerization reaction via Stille coupling polycondensation between 2,6-bis(trimethytin)-4,8-bis(5-(2-ethylhexyl)thiophen-2-yl)benzo[1,2-b:4,5-b']dithiophene (118, 88.5, 59, 29.5, 0 mg, respectively) and 1,3-bis(5-bromothiophen-2-yl)-5,7-bis(2-ethylhexyl)benzo[1,2-c:4,5-c']dithiophene-4,8-dione (100 mg), affording PBDB-TF*x* (Supplementary Scheme 1, x = 0, 0.25, 0.5, 0.75, 1, respectively). Pd2(dba)3 (2 mol% vs. Y6-OD-2Br) and P(o-tolyl)3 (8 mol% vs. Y6-OD-2Br) were added to the chlorobenzene solution (15 mL) containing the monomers, and the rection mixtures were stirred 120 °C for 48 h. These polymers share the same solubilizing side-chains to ensure good solubility, while the backbone fluorine substitution is expected to fine-tune the frontier orbital energy levels and the polymer:polymer blend thin films morphology. High-temperature gel permeation chromatography (HT-GPC) at 150 °C using 1,2,4-tricholorobenzene as the eluent and polystyrene as a standard showed comparable average molecular weights (M_n) around 80 kDa and polydispersity index (PDI) < 2.8, which minimize the effects of molecular weight on the polymer properties. The increasing ratio of the fluorine-containing monomer in **PBDB-TF*x*** has also been confirmed by well-resolved FT-IR in

the solid state (Supplementary Figure 2). Because the ratios precisely follow the feed ratio in the range of the integration error of the instrumentation, we can assume the repeating units to be mainly statistically distributed in the polymer.

Supplementary Figures

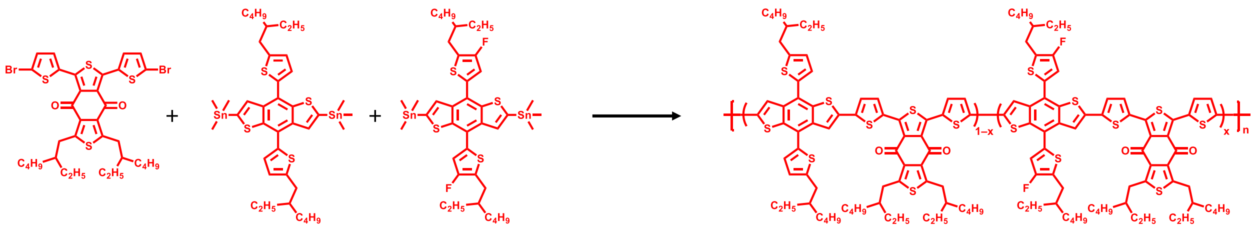

Supplementary Figure 1. Synthesis route of **PBDB-TF*x*** (x = 0, 0.25, 0.5, 0.75, 1).

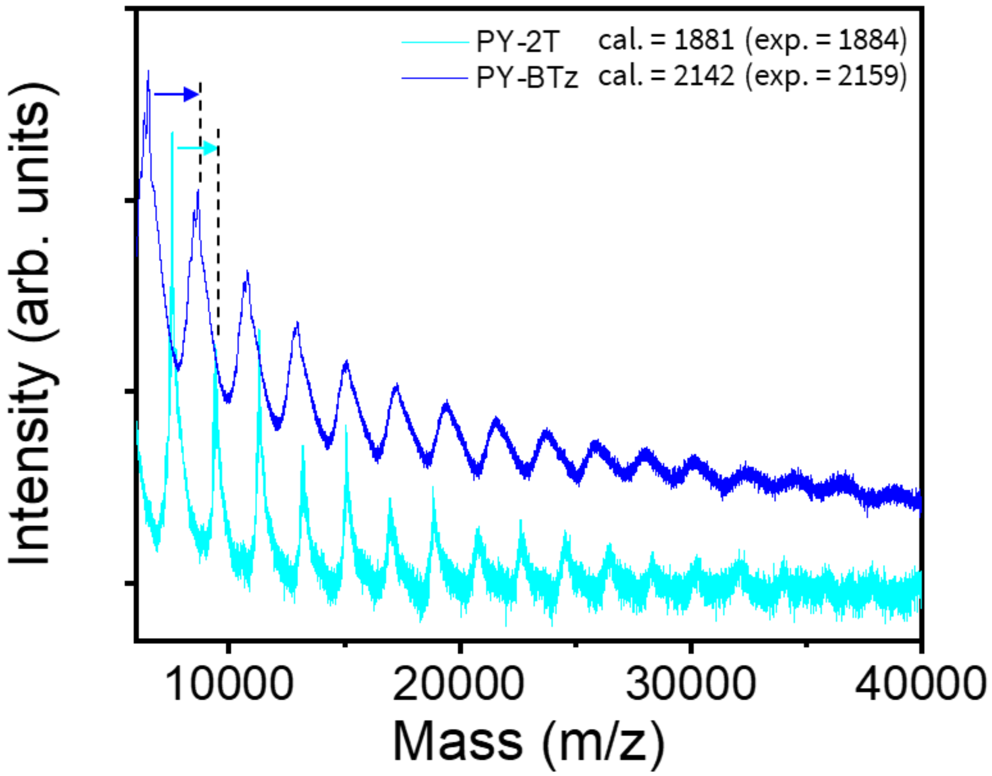

Supplementary Figure 2. MALDI-TOF signal in negative mode of **PY-BTz** and **PY-2T**. The experimental peaks agree with the calculated repeat units of **PY-BTz** (2159 g/mol) and **PY-2T** (1884 g/mol).

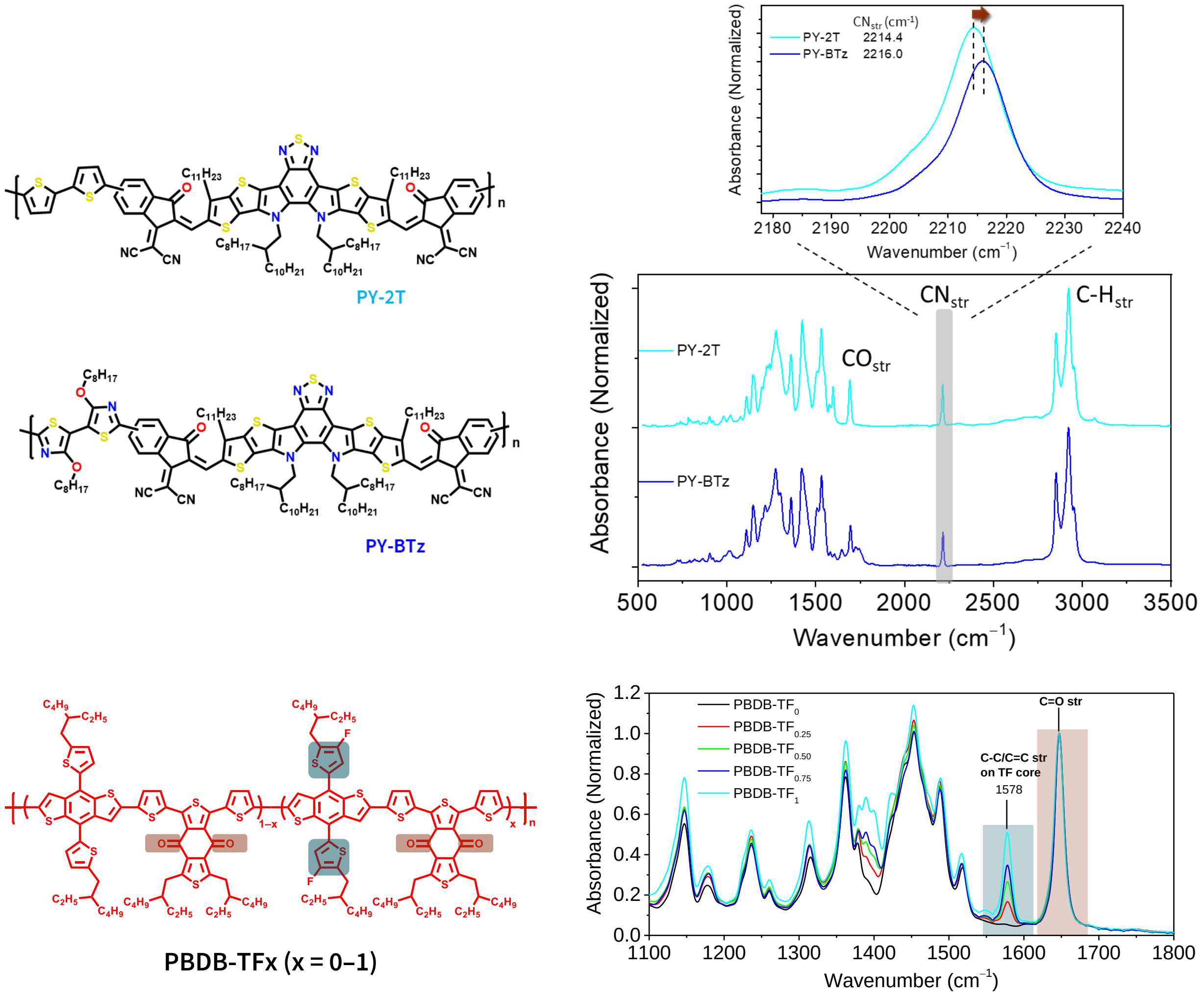

Supplementary Figure 3. ATR-FTIR spectra of **PY-BTz**, **PY-2T**, and **PBDB-TF$_x$** drop-cast films.

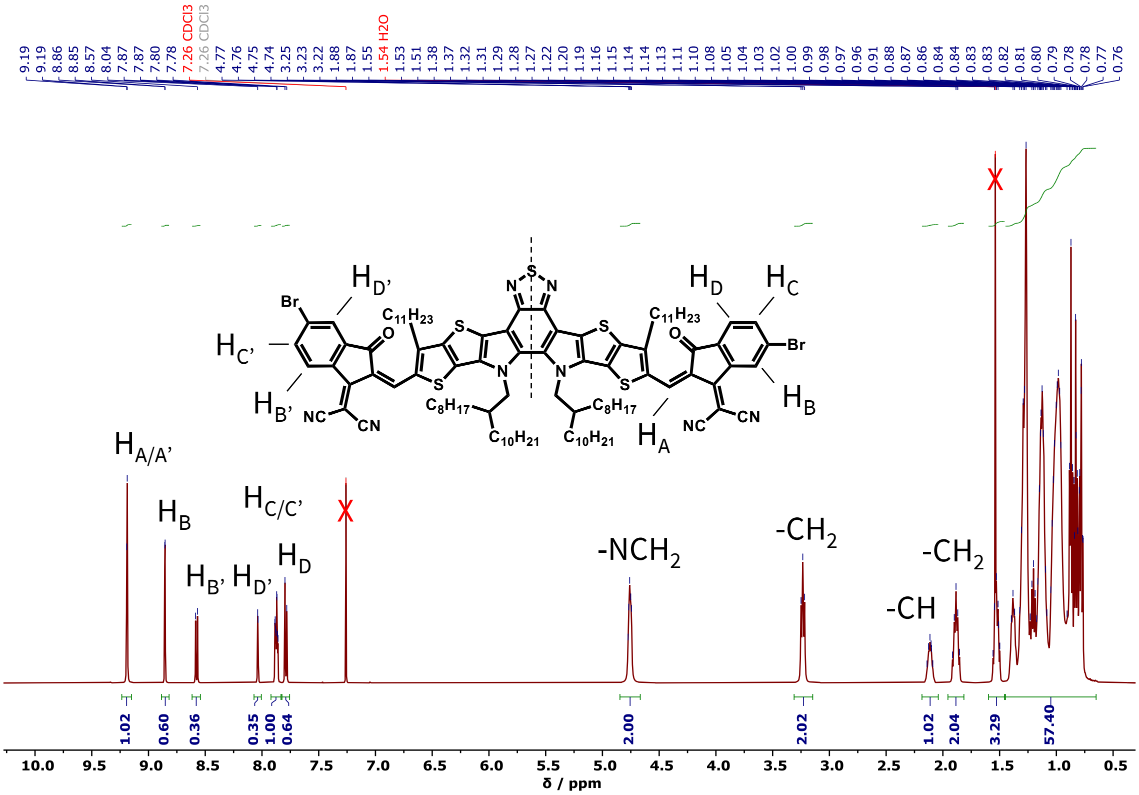

Supplementary Figure 4. ^{1}H NMR spectrum of Y6-OD-2Br in $CDCl_3$.

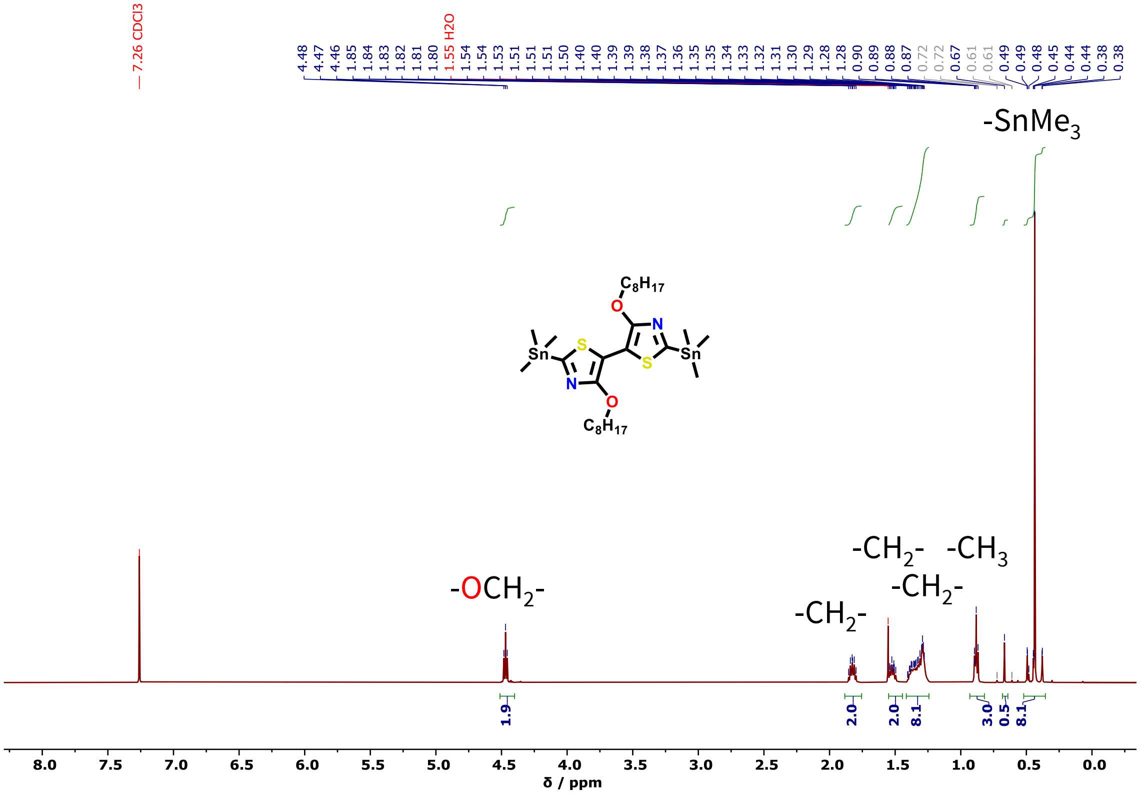

Supplementary Figure 5. ^{1}H NMR spectrum of 2,2'-bis(trimethylstannyl)-5,5'-bithiazole in $CDCl_3$.

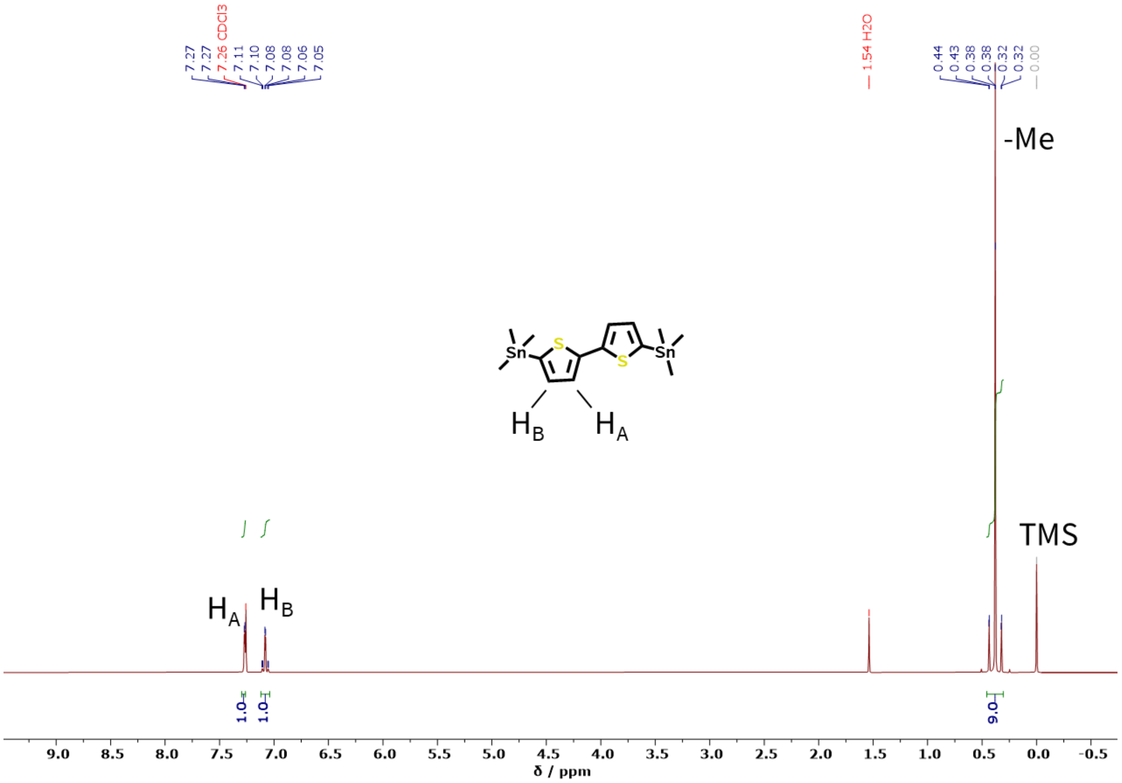

Supplementary Figure 6. ^{1}H NMR spectrum of 5,5′-bis(trimethylstannyl)-2,2′bithiophene in $CDCl_3$.

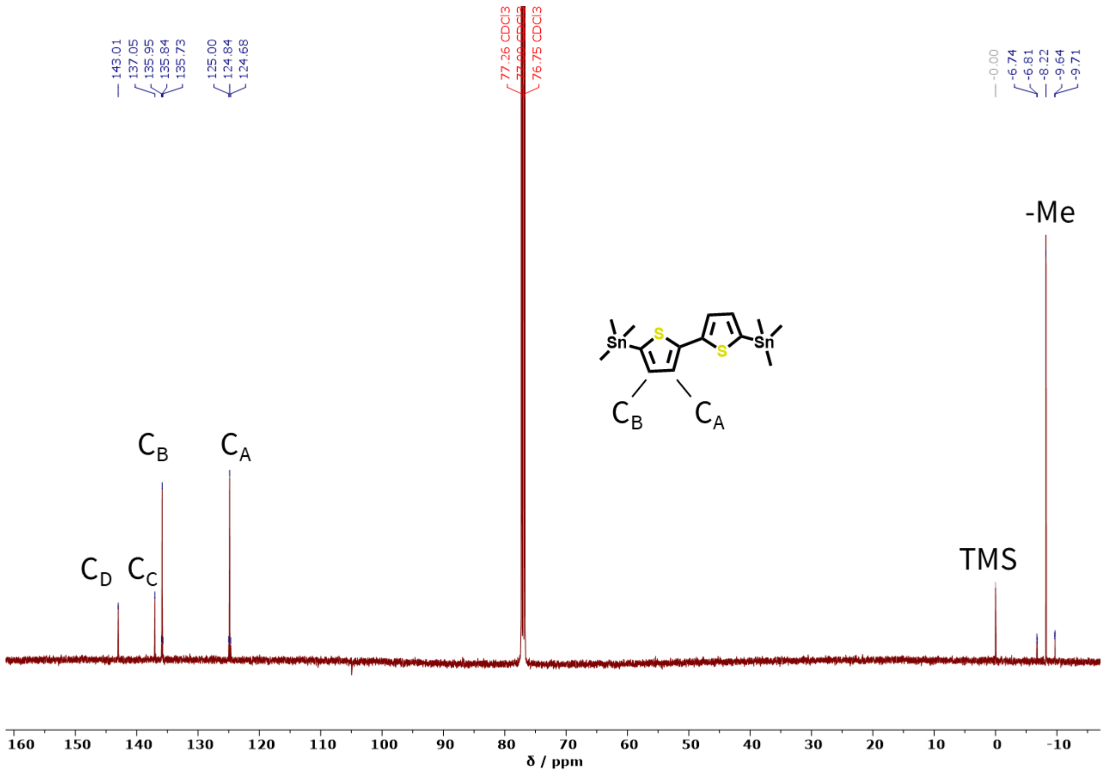

Supplementary Figure 7. ^{13}C NMR spectrum of 5,5′-bis(trimethylstannyl)-2,2′bithiophene in $CDCl_3$.

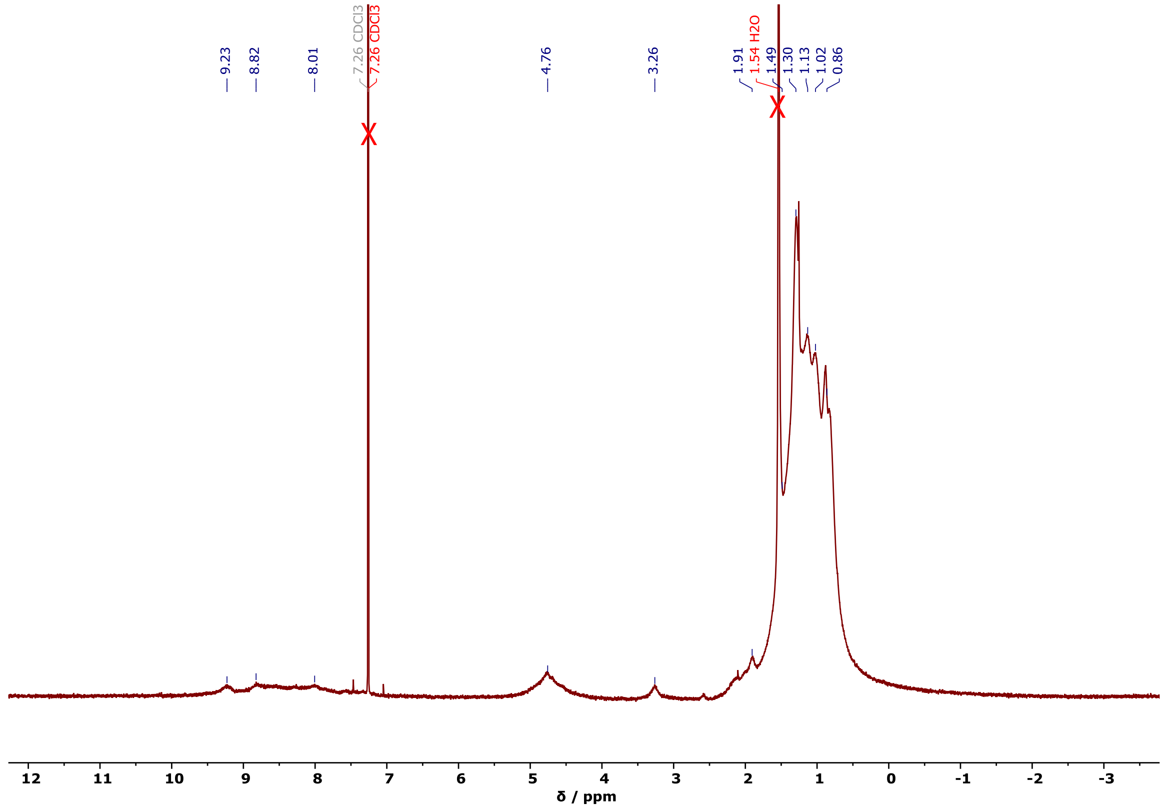

Supplementary Figure 8. ^{1}H NMR spectrum of **PY-BTz** in $CDCl_3$.

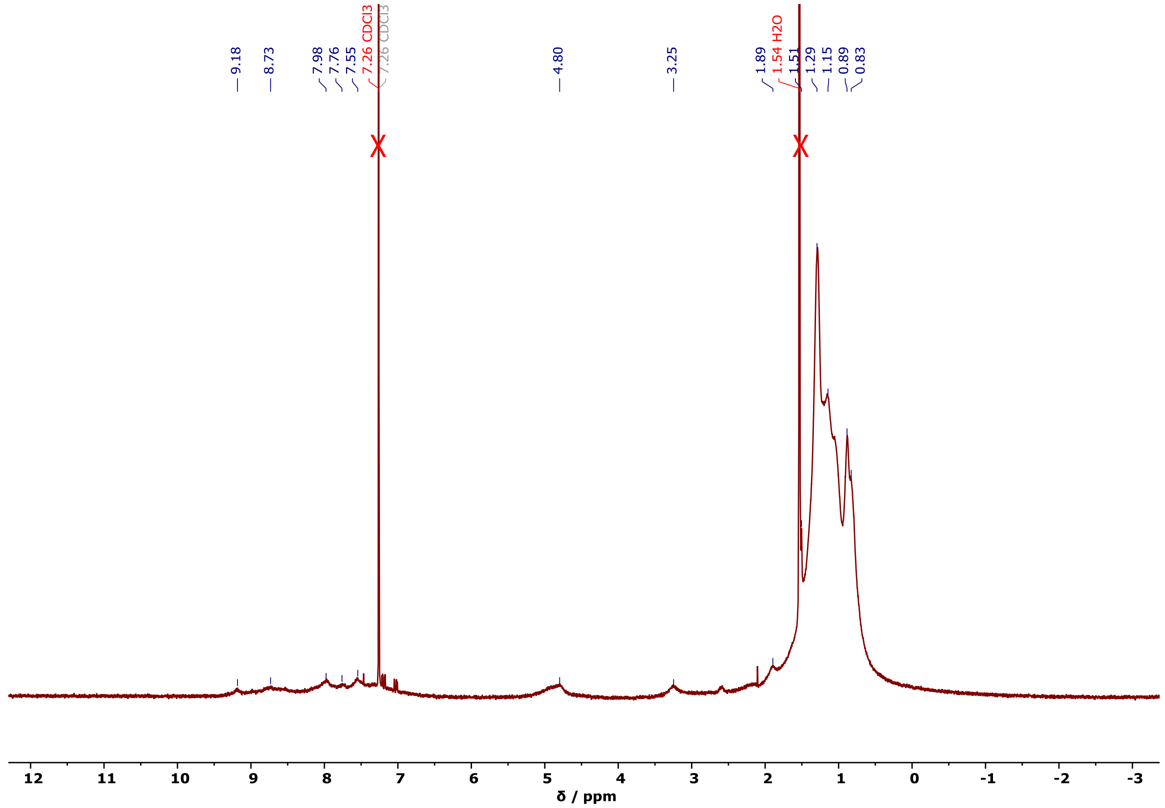

Supplementary Figure 9. ^{1}H NMR spectrum of **PY-2T** in $CDCl_3$.

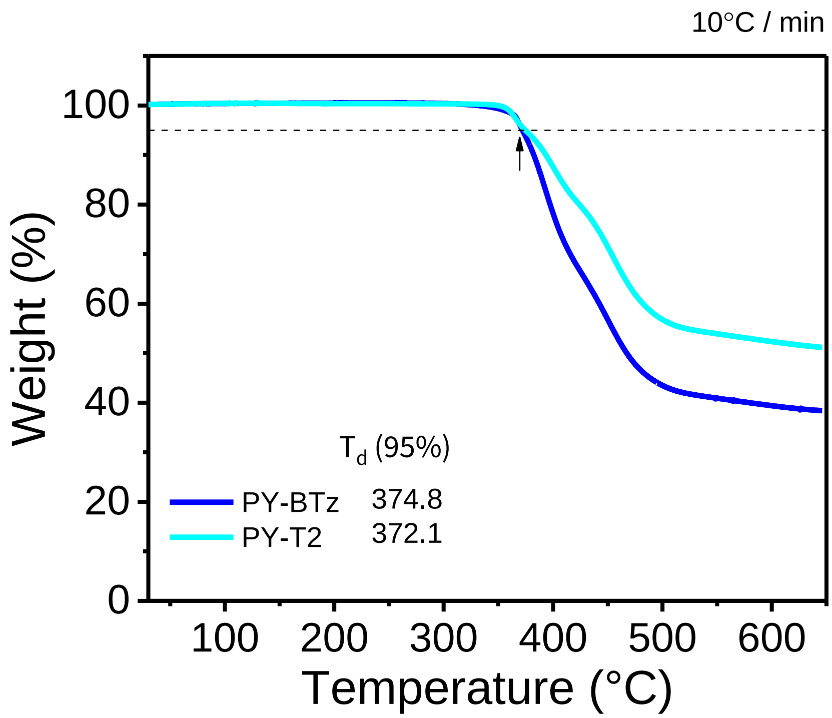

Supplementary Figure 10. TGA curves of **PY-BTz** and **PY-2T** at the rate of 10 °C/min.

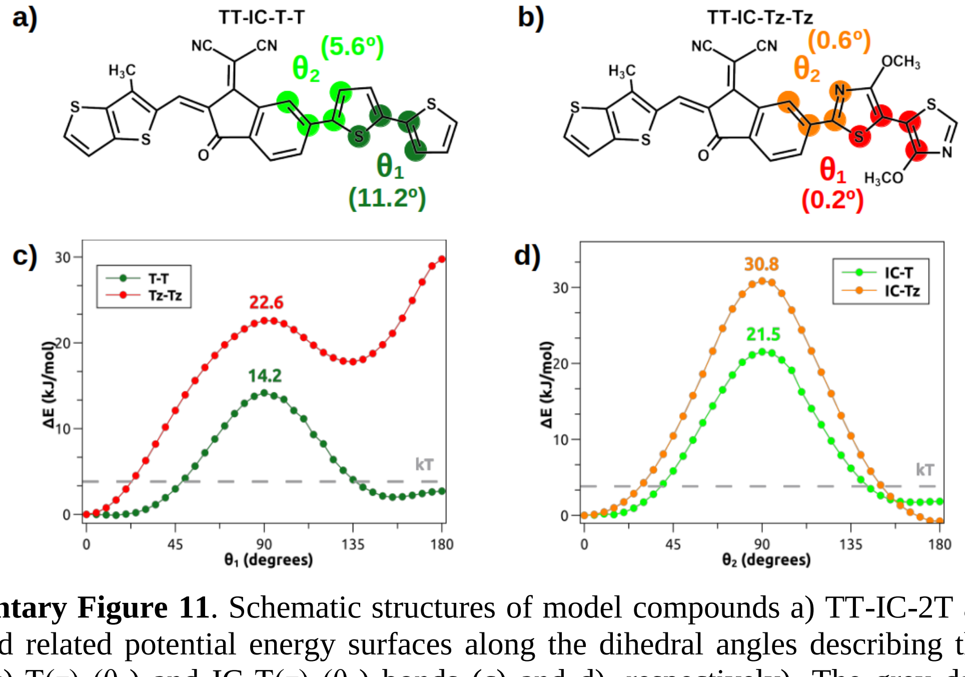

Supplementary Figure 11. Schematic structures of model compounds a) TT-IC-2T and b) TT-IC-BTz and related potential energy surfaces along the dihedral angles describing the rotation around T(z)-T(z) (θ_1) and IC-T(z) (θ_2) bonds (c) and d), respectively). The grey dashed lines indicate thermal energy at 298 K.

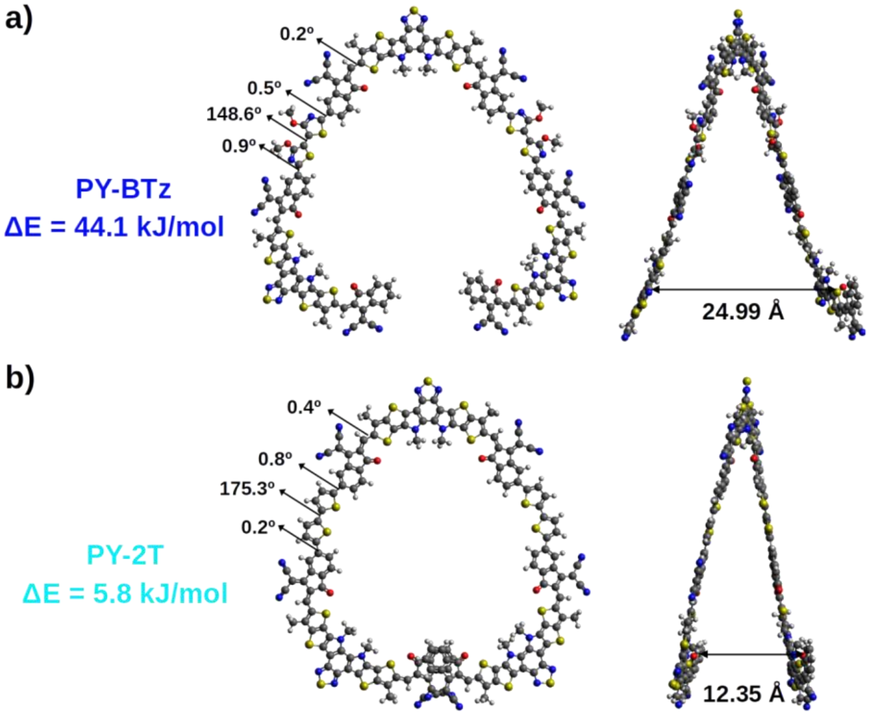

Supplementary Figure 12. Front (left) and side (right) view of optimized neutral ground-state coiled structures of the trimers of the repeating units of a) PY-BTz and b) PY-2T Red: oxygen atoms; yellow: sulfur atoms; blue: nitrogen atoms. The most relevant geometrical parameters and the relative energies with respect to the optimized planar zig-zag conformers are reported.

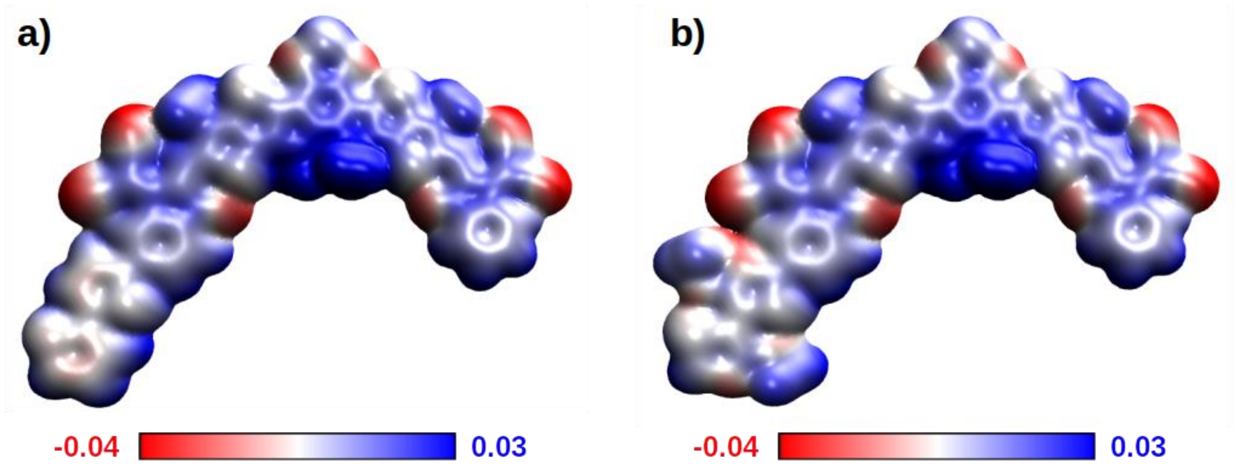

Supplementary Figure 13. Plots of selected kinetic traces superimposed with matching curves at different wavelengths— 3D maps of the Molecular Electrostatic Potential (MEP) at the 1 me/a_0^3 isodensity surface of the a) PY-2T and b) PY-BTz monomers. The value of the potential on both surfaces ranges from -0.04 Hartree/e (left side of the bottom bar) to 0.03 Hartree/e (right side of the bottom bar).

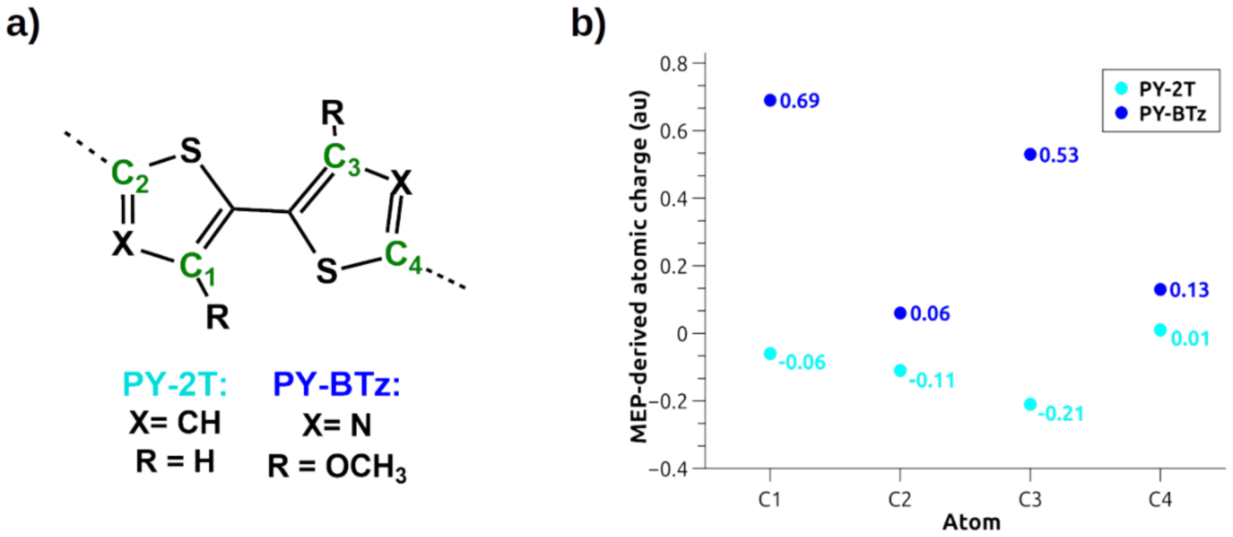

Supplementary Figure 14. a) Schematic structure of the π-linker in PY-2T and PY-BTz monomers. b) Molecular electrostatic potential-derived (MEP-derived) atomic charges of selected carbon atoms of bithiophene and bithiazole units in PY-2T and PY-BTz, respectively.

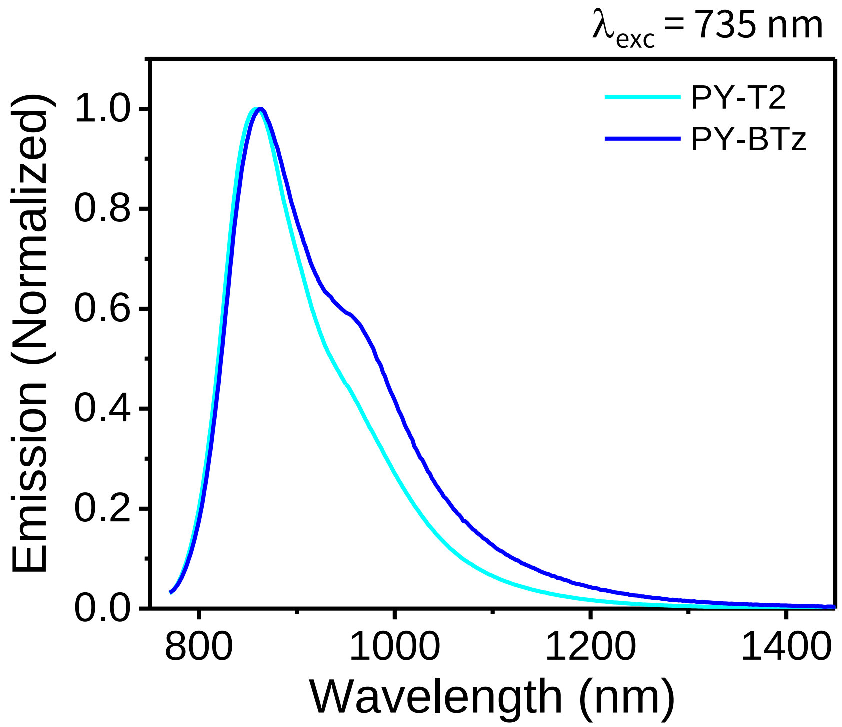

Supplementary Figure 15. PL spectra of **PY-BTz** and **PY-2T** thin films on glass substrate excited at 735 nm.

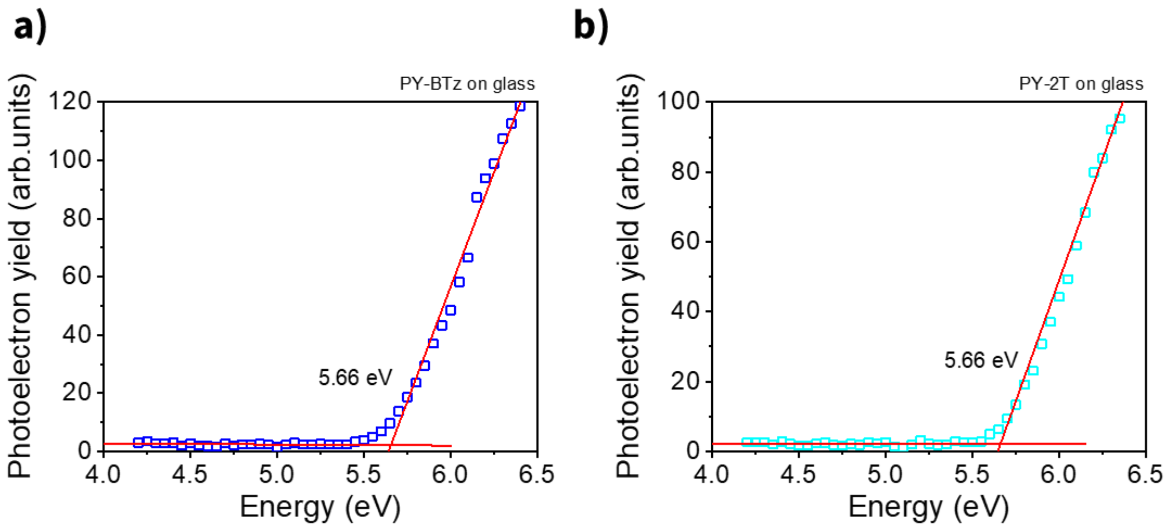

Supplementary Figure 16. PESA spectra of a) **PY-BTz** and b) **PY-2T** thin films on glass.

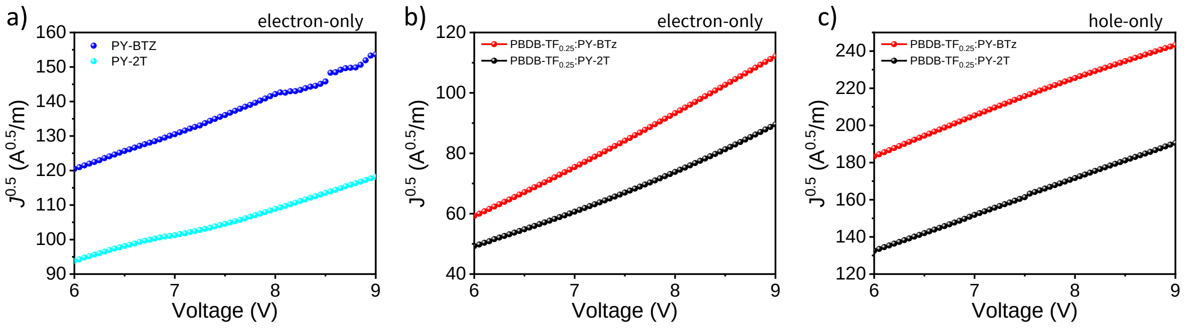

Supplementary Figure 17. $J^{1/2}$-V characteristics of a) electron-only devices of **PY-BTz** and **PY-2T** films. $J^{1/2}$-V characteristics of b) electron-only devices and c) hole-only devices of the PBDB-$TF_{0.25}$:**PY-BTz** and PBDB-$TF_{0.25}$:**PY-2T**.

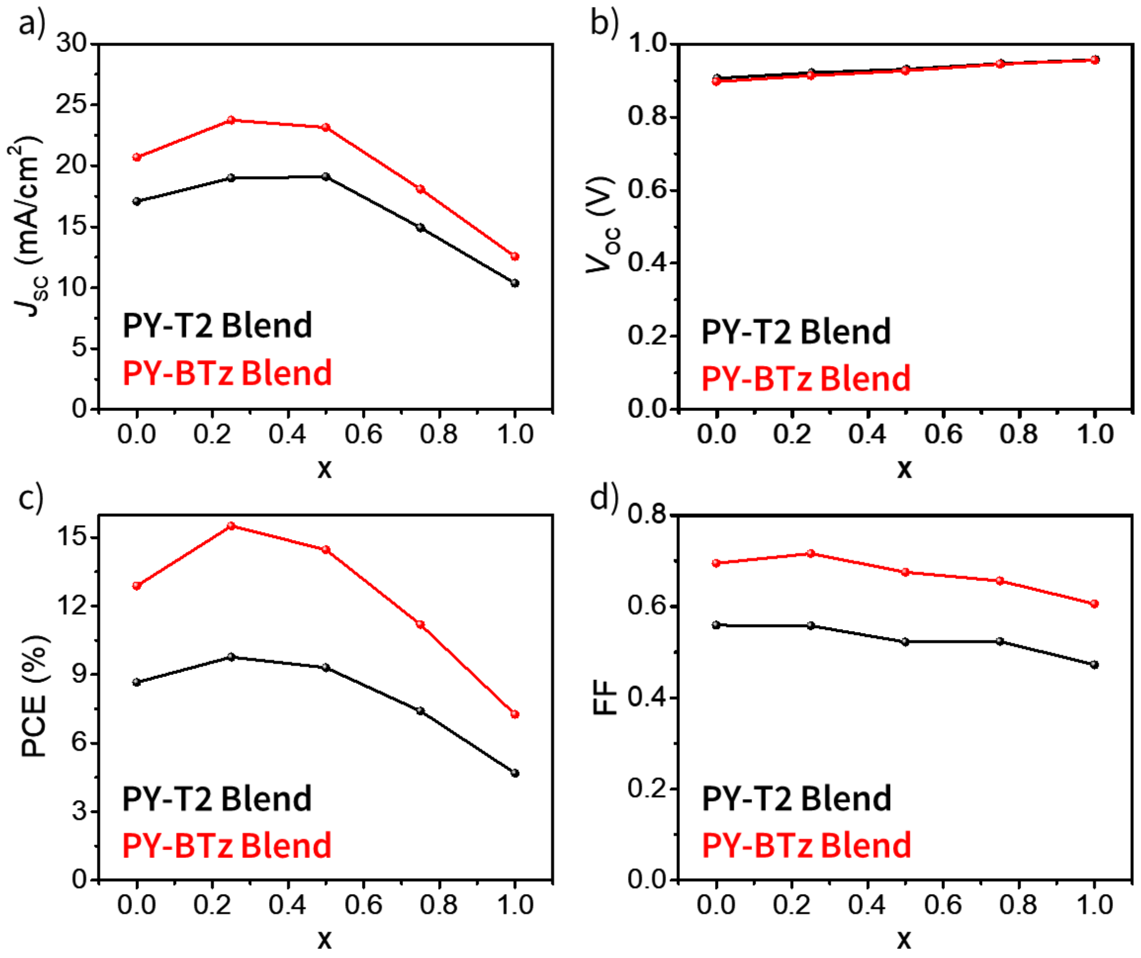

Supplementary Figure 18. a) Short-circuit current density, b) open-circuit voltage, c) fill factor, and d) PCE as a function the five **PBDB-TF$_x$** (x = 0, 0.25, 0.5, 0.75, 1) polymer donors. The average values are from 4 individual devices.

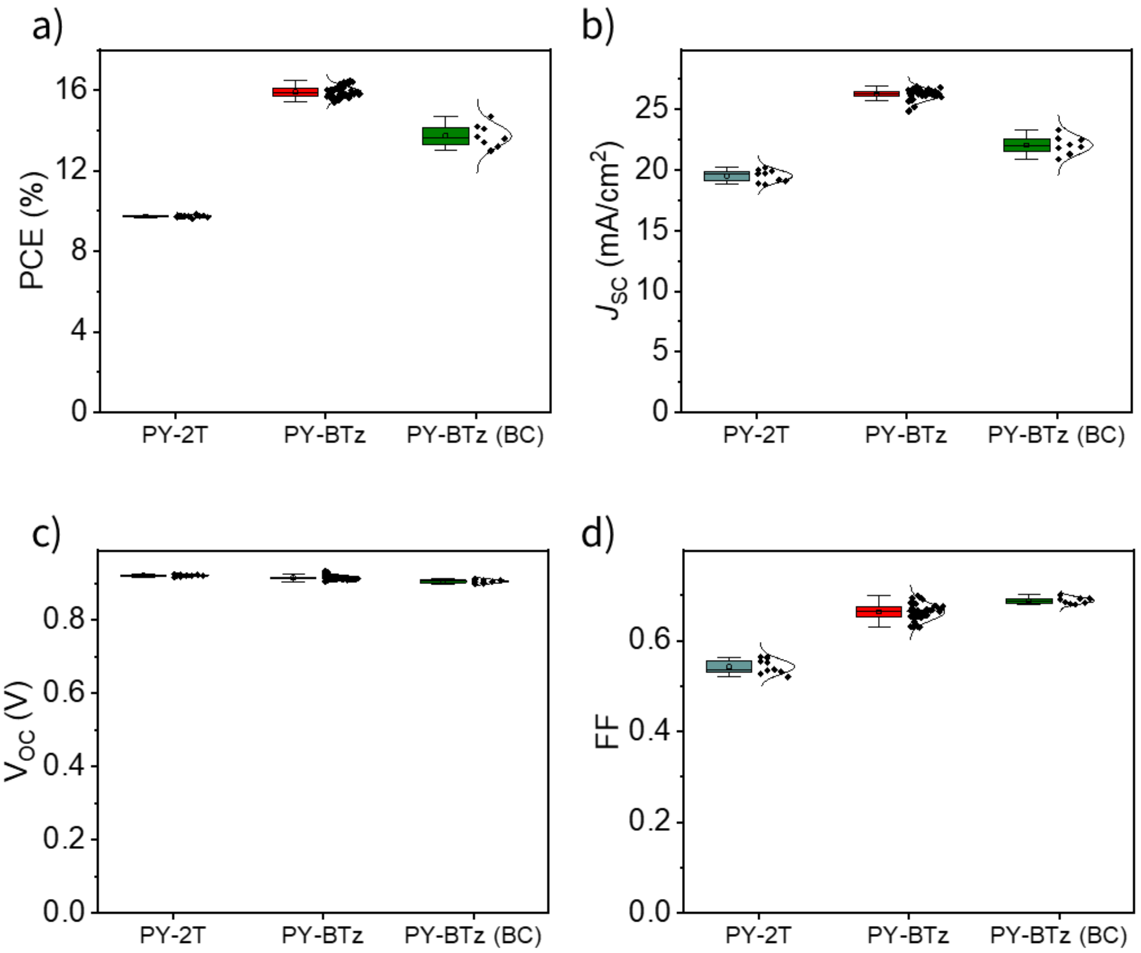

Supplementary Figure 19. Statistical histograms of a) PCEs, b) J_{SC}, c) V_{OC}, and d) FF of all-PSCs based on PBDB-TF$_{0.25}$:**PY-2T**, PBDB-TF$_{0.25}$:**PY-BTz**, and PBDB-TF$_{0.25}$:**PY-BTz** fabricated with blade coating. The averages are from 9, 43, and 8 individual devices (over 2 batches), respectively.

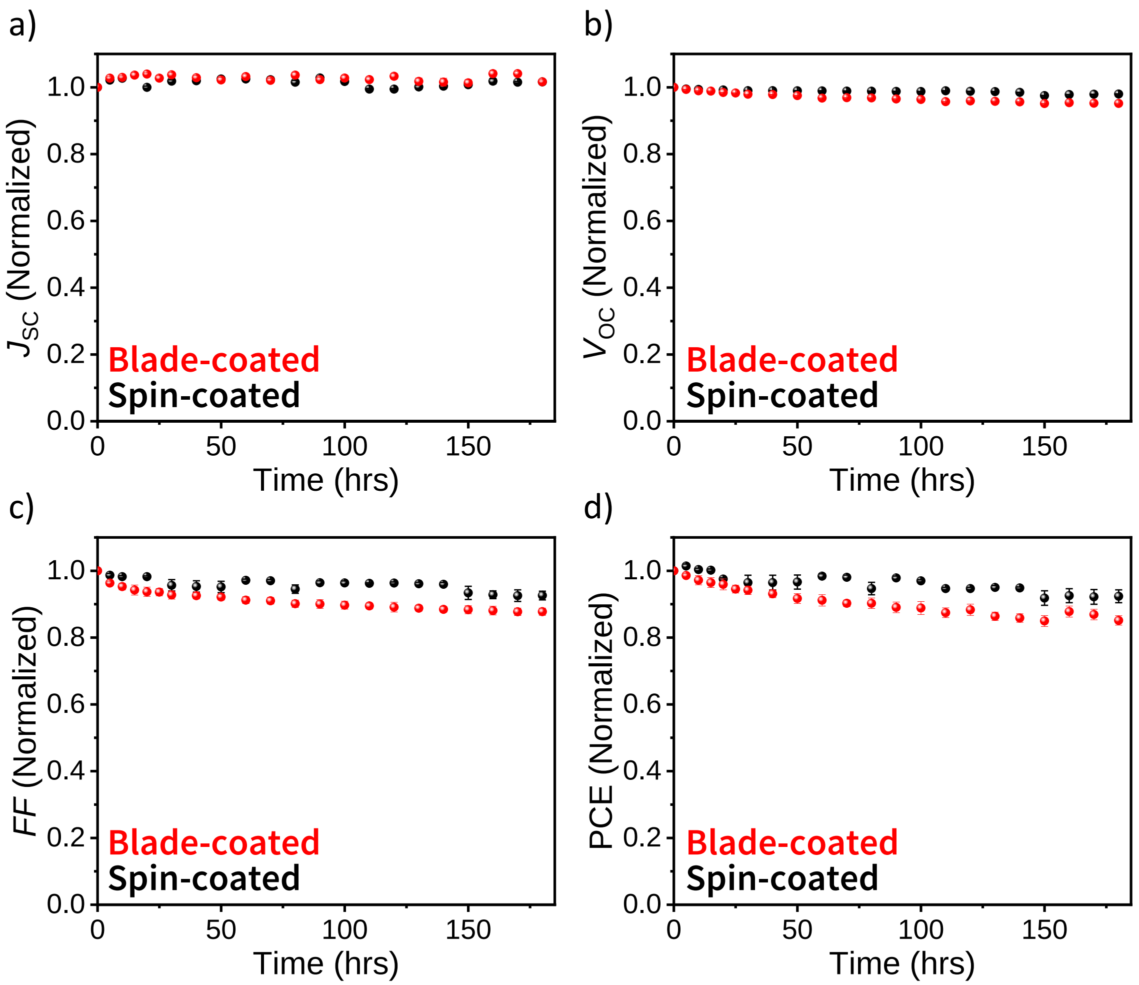

Supplementary Figure 20. a) Normalized short-circuit current density, b) open-circuit voltage, c) fill factor, and d) PCE as a function of annealing time for spin-coated and blade-coated all-PSCs based on PBDB-TF$_{0.25}$:**PY-BTz** baked on a hotplate at 90 °C in a nitrogen glove box. The averages are from 5 individual devices.

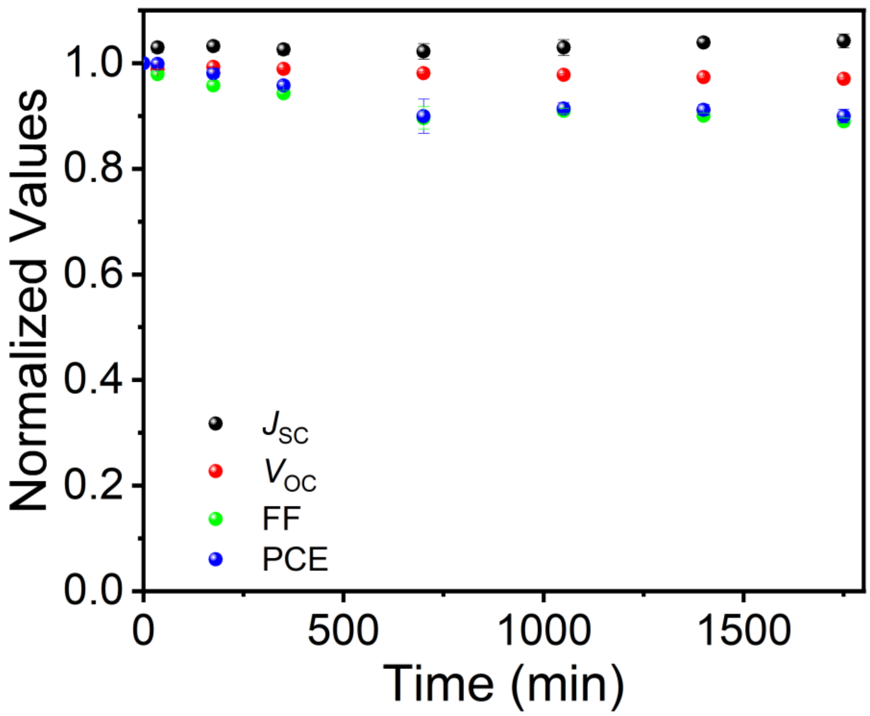

Supplementary Figure 21. Normalized short-circuit current density, open-circuit voltage, fill factor, and PCE as a function of annealing time for blade-coated all-PSCs based on PBDB-$TF_{0.25}$:**PY-BTz** under continuous illumination (100 mWcm^{-2}) in a nitrogen glove box. The averages are from 5 individual devices.

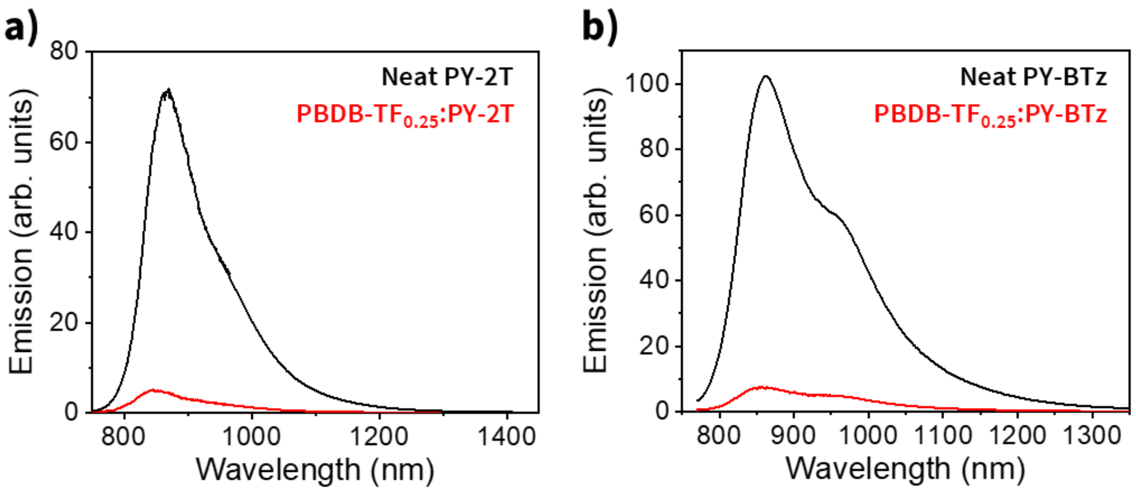

Supplementary Figure 22. PL spectra of neat a) **PY-2T** and b) **PY-BTz** thin films and corresponding blend films with PBDB-$TF_{0.25}$ on glass substrate excited at 735 nm.

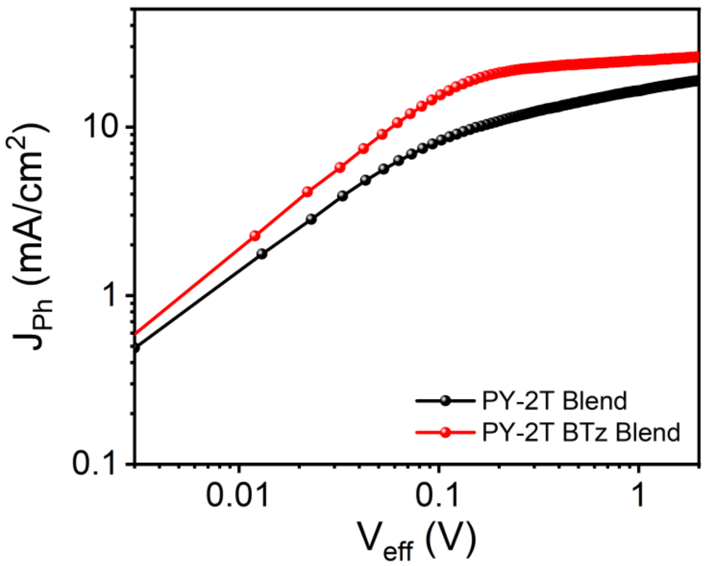

Supplementary Figure 23. J_{Ph}-V_{eff} curves of all-PSCs based on PBDB-TF$_{0.25}$:**PY-BTz** and PBDB-TF$_{0.25}$:**PY-2T**.

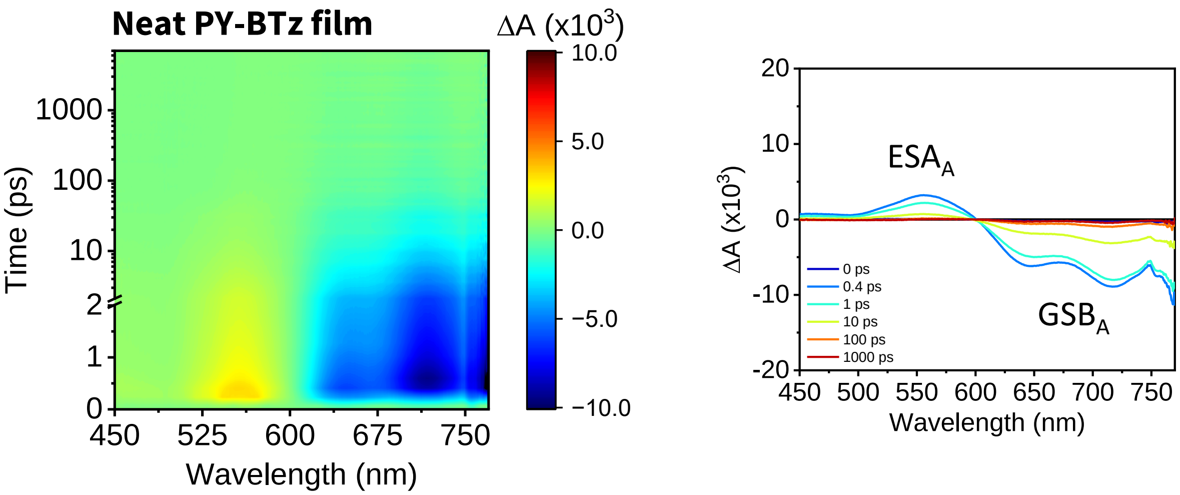

Supplementary Figure 24. Femtosecond transient absorption spectra of neat **PY-BTz** film by exciting at 800 nm. Two samples were measured showing consistent results.

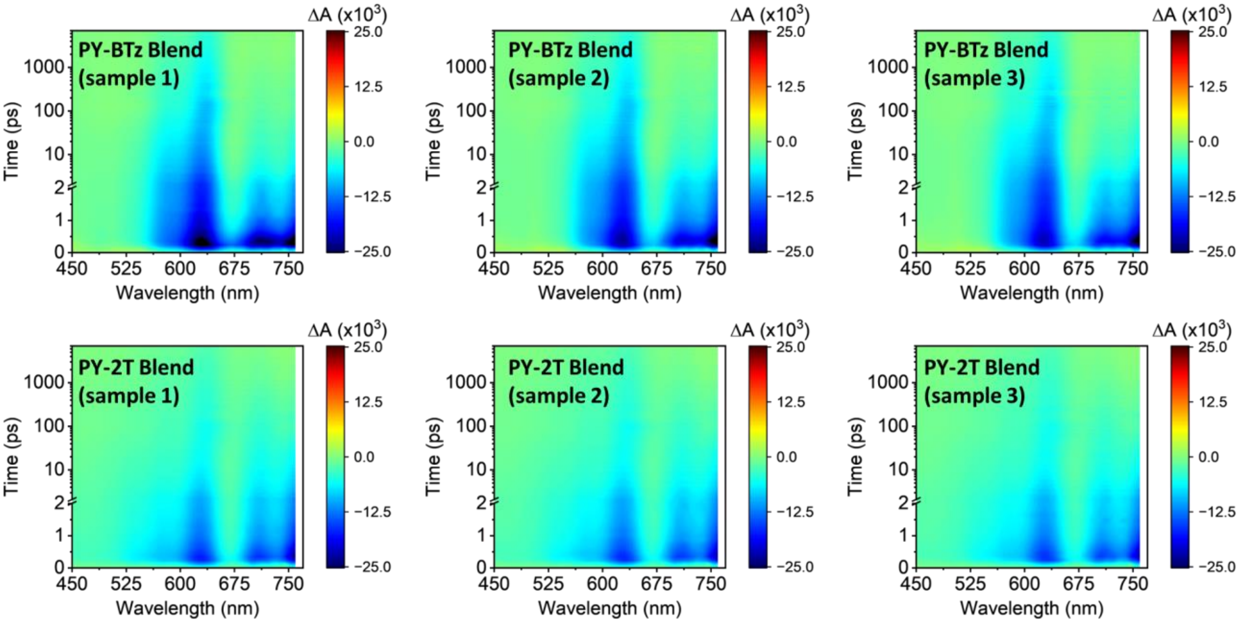

Supplementary Figure 25. Femtosecond transient absorption spectra of **PY-BTz** and **PY-2T** based blend films film by exciting at 800 nm. Three samples for each system were measured showing consistent results.

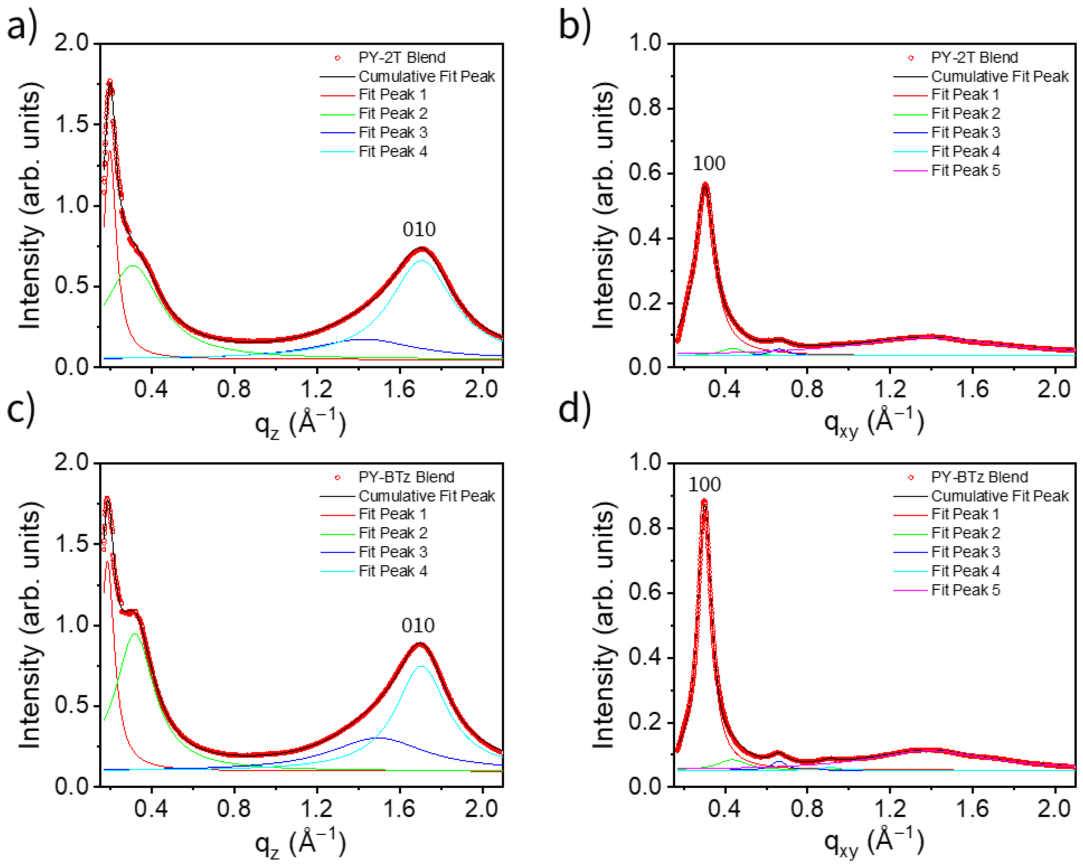

Supplementary Figure 26. Fittings of in-plane and out-of-plane scattering profiles of GIWAXS for a-b) PBDB-TF$_{0.25}$:**PY-2T** and c-d) PBDB-TF$_{0.25}$:**PY-BTz** films.

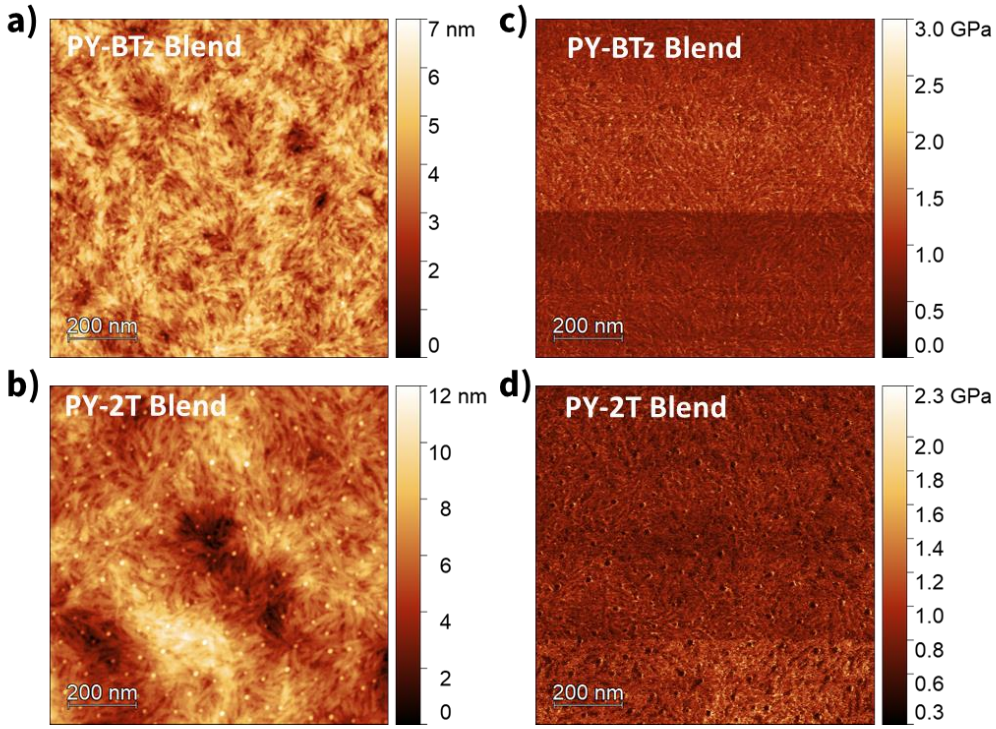

Supplementary Figure 27. a-b) AFM height images (1000 × 1000 nm) and c-d) corresponding DMT (Derjaguin, Muller, Toporov) modulus image of blended thin film measured in peak force quantitative nanomechanical (QNM) mode.

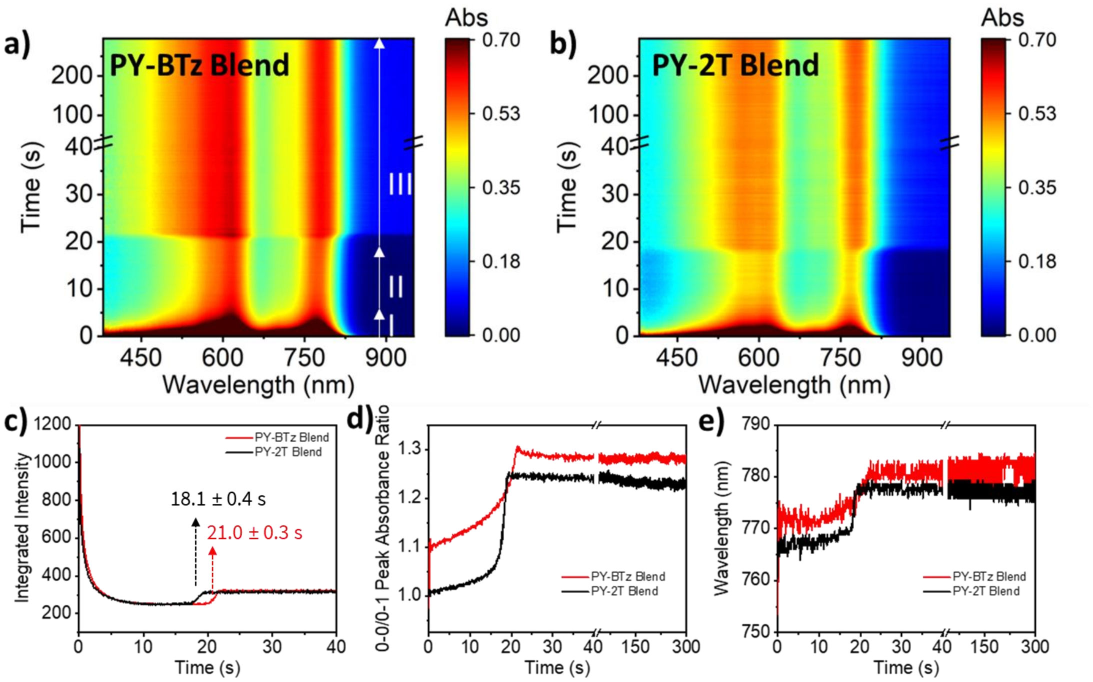

Supplementary Figure 28. a-b) In-situ UV-vis absorption spectra during spin-coating of all-PSCs based on PBDB-TF$_{0.25}$:**PY-BTz** and PBDB-TF$_{0.25}$:**PY-2T**. Morphology evolution kinetics by tracking changes in c) absorbance of the peaks, d) ratio of 0-0 and 0-1 peaks of the acceptor polymer, and e) wavelength of 0-0 peaks.

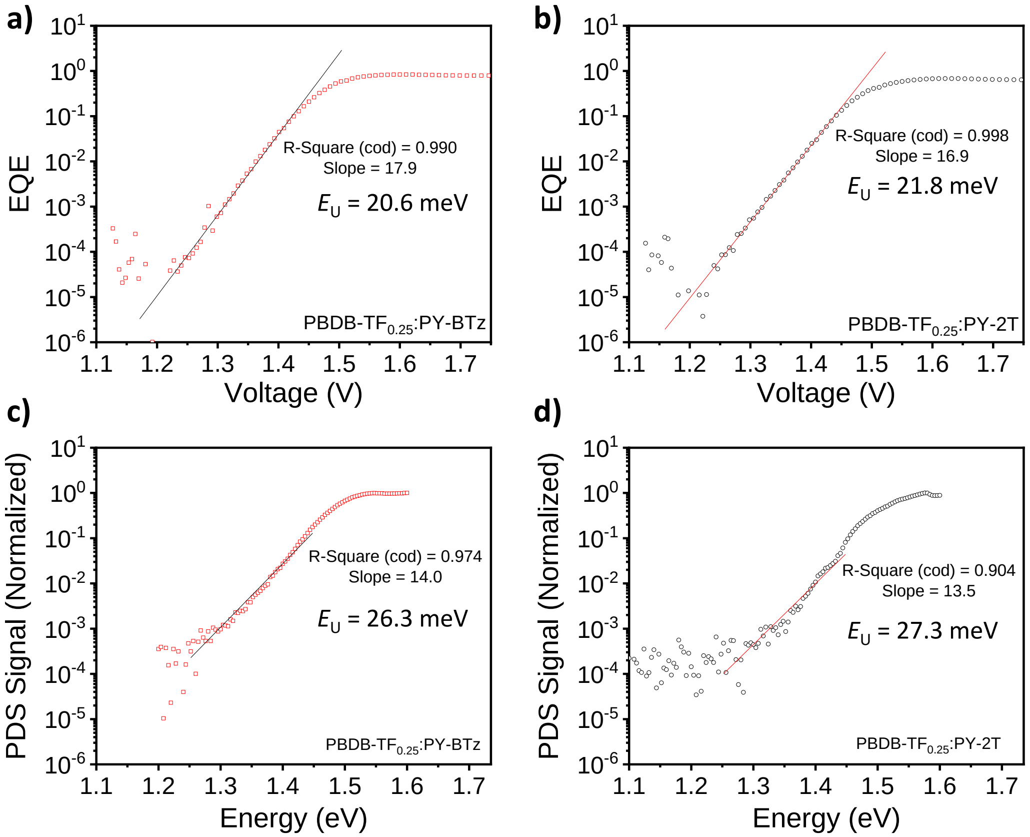

Supplementary Figure 29. a-b) EQE and c-d) PDS of the all-PSCs based on PBDB-TF$_{0.25}$:**PY-BTz** and PBDB-TF$_{0.25}$:**PY-2T** in the low-energy region. Two samples were measured for each sample.

Supplementary Tables

Supplementary Table 1. Batch-to-batch variation analysis of photovoltaic parameters of the All-PSC devices based on PBDB-$TF_{0.25}$:PY-BTz under the illumination of AM 1.5 G, 100 mW cm^{-2}.

Acceptor	Batch no.	Scale (mg)	M_n (kDa)	$Đ_M$	J_{sc} (mA/cm^2)	V_{OC} (V)	Fill Factor	PCE_{ave} (%)
**PY-BTz**	1	60	15.5	2.64	26.5 ± 0.3	0.921 ± 0.007	0.646 ± 0.01	15.7 ± 0.2
**PY-BTz**	2	60	14.2	2.59	26.1 ± 0.5	0.913 ± 0.002	0.673 ± 0.01	16.0 ± 0.2

[a]The average values with standard deviations were obtained 15 (batch no 1) and 28 samples (batch no 2). Device structure of ITO/ZnO/PFNBr/Active-layer/MoO_3/Ag. Device area: 4 mm^2.

Supplementary Table 2. Reported All-PSCs with high thermal stability.

Entry no.	Donor	Acceptor	Morphology	Initial PCE (%)	T (°C)	Time (h)	PCE Retention (%)	Reference
1	PBDT-TAZ	NOE10	Binary BHJ	8.1	65	300	97	*J. Am. Chem. Soc. 2018,* ***140****, 8934-8943*
2	PM6	PF1-TS4	Binary BHJ	8.63	80	180	70	*Angew. Chem. Int. Ed. 2020,* ***59****, 19835-19840*
3	PBDBT-BV20	N2200-TV10	Binary BHJ (crosslinked)	9.24	80	90	91	*ACS Appl. Mater. Interfaces 2021,* ***13****, 16754-16765*
4	PM6	PY2F-T:PYT	Ternary BHJ	17.2	65	300	83.5	*Joule 2021,* ***5****, 1548-1565*
5	PBDB-T	PTCloY	Binary BHJ	12.74	80	160	87	*Sci China Chem 2022,* ***65****, 182-189*
6	PBDB-T	PYT-H	Binary BHJ	14.29	80	408	96.96	*J. Mater. Chem. C 2022,* ***10****, 1850-1861*
7	PM6:PM6TPO	PY-IT	Ternary BHJ	17.0	80	96	85	*Adv. Sci. 2022,* ***9****, 2204030*

Supplementary Table 3. Peak positions and coherence lengths of the optimized All-PSC active layer based on PBDB-$TF_{0.25}$:PY-BTz and PBDB-$TF_{0.25}$:PY-2T.

Samples	q(100) [1/Å]	Lamellar spacing [Å]	100 peak FWHM [1/Å]	100 peak Coherence length [Å]	q(010) [1/Å]	π-π spacing [Å]	010 peak FWHM [1/Å]	010 peak Coherence length [Å]
PY-2T Blend	0.3018±0.0001	20.81±0.07	0.114±0.002	50±0.9	1.706±0.004	3.68±0.01	0.371±0.002	15.2±0.1
PY-BTz Blend	0.2998±0.0001	20.95±0.07	0.081±0.002	70±1.7	1.706±0.004	3.68±0.01	0.307±0.002	18.4±0.1

Supplementary Table 4. Selected high-performing spin-coated binary all-PSCs.

Entry no.	J_{sc} (mA/cm2)	V_{OC} (V)	Fill Factor (%)	PCE (%)	Reference
1	0.83	14.18	70.24	8.27	*Adv. Mater.*, 2016, **28**, 1884-1890
2	0.84	14.4	74	9	*Energy Environ. Sci.*, 2017, **10**, 2212-2221
3	0.849	15.17	70.36	9.16	*Energy Environ. Sci.*, 2017, **10**, 1243-1251
4	0.87	15.57	73.39	10.1	*Adv. Mater.*, 2017, **29**, 1703906
5	0.83	16.05	68.99	9.19	*Angew. Chem. Int. Ed.*, 2017, **56**, 13503-13507
6	0.74	17.07	67	8.59	*Adv. Mater.*, 2017, **29**, 1700309
7	0.884	12.91	75.4	8.61	*ACS Appl. Mater. Interfaces*, 2018, **10**, 38302-38309
8	0.87	14.5	67.8	8.23	*J. Mater. Chem. A*, 2018, **6**, 10421-10432
9	0.87	14.27	63.02	8	*Sci China Chem*, 2018, **61**, 576-583
10	0.85	14.83	64.32	8.13	*Angew. Chem. Int. Ed.*, 2018, **57**, 4580-4584
11	0.85	14.89	75.65	9.56	*Adv. Energy Mater.*, 2018, **8**, 1703085
12	0.86	15.8	73	10	*Adv. Mater.*, 2018, **30**, 1803166
13	0.84	15.77	74.98	10.09	*Sol. RRL*, 2018, **2**, 1800196
14	0.85	18.32	57	9.38	*Chem. Mater.*, 2018, **30**, 6540-6548
15	1.1	11.3	61	8	*Adv. Energy Mater.*, 2018, **8**, 1700908
16	0.84	12.9	75	8.1	*J. Am. Chem. Soc.*, 2018, **140**, 8934-8943
17	0.9	13.5	67	8.1	*J. Mater. Chem. A*, 2018, **6**, 16403-16411
18	0.9	14.2	65	8.32	*Adv. Mater.*, 2019, **31**, 1905161
19	1.05	13.56	58.25	8.28	*Adv. Funct. Mater.*, 2019, **29**, 1903970
20	0.87	14.72	64.1	8.21	*Adv. Funct. Mater.*, 2019, **29**, 1806747
21	0.89	14.24	69	8.63	*Sol. RRL*, 2019, **3**, 1800340
22	0.84	14.86	66.65	8.36	*Nano Energy*, 2019, **59**, 277-284
23	1	15.2	69	10.5	*J. Mater. Chem. A*, 2019, **7**, 16190-16196
24	0.83	15.3	70	9.1	*Sol. RRL*, 2019, **3**, 1900032
25	0.96	15.27	68	10.3	*ACS Energy Lett.*, 2019, **4**, 417-422
26	0.904	15.33	68.7	9.52	*ACS Energy Lett.*, 2019, **4**, 2277-2286
27	1.03	14.88	58.46	8.98	*Sol. RRL*, 2019, **3**, 1900107
28	0.85	16.5	77.9	11	*Energy Environ. Sci.*, 2019, **12**, 157-163
29	0.78	16.77	68.07	9.03	*Sci China Chem*, 2019, **62**, 238-244
30	0.85	17.2	77.9	11.5	*Nano Energy*, 2019, **64**, 103931
31	0.88	17.62	75.78	11.76	*Adv. Mater.*, 2019, **31**, 1902899
32	0.82	17.52	72.1	10.4	*Nat. Commun.*, 2019, **10**, 4100
33	0.86	18.55	64	10.1	*ACS Energy Lett.*, 2019, **4**, 1162-1170
34	0.96	17.1	68.2	11.2	*Sci China Chem*, 2019, **62**, 845-850
35	0.926	13.012	69.8	8.776	*Adv. Mater.*, 2019, **31**, 1904585
36	1.05	13.6	56.5	8.1	*Adv. Mater.*, 2019, **31**, 1807220
37	0.99	16.48	66.1	10.77	*Joule*, 2020, **4**, 658-672
38	0.9	22.6	71	14.4	*Nano Energy*, 2020, **72**, 104718

39	0.88	15.9	61	8.5	*J. Am. Chem. Soc.*, 2020, **142**, 392-406
40	1.17	13.39	64	10.07	*Chem. Mater.*, 2020, **32**, 1308-1314
41	0.93	21.78	66.33	13.44	*Joule*, 2020, **4**, 1070-1086
42	0.943	15.75	68.2	10.13	*Sol. RRL*, 2020, 2000142
43	0.899	21.33	65.3	12.52	*Angew. Chem. Int. Ed.*, 2020, **59**, 15181-15185
44	0.96	20.6	72.1	14.3	*Adv. Mater.*, 2020, 2004183
45	0.946	20.65	74	14.45	*Energy Environ. Sci.*, 2020, **13**, 5017-5027
46	0.933	22.3	72.3	15.05	*Adv. Mater.*, 2020, **32**, 2005942
47	0.87	23.96	72.67	15.11	*ACS Energy Lett.*, 2020, **5**, 3702-3707
48	0.88	14.41	66	8.21	*Chem. Mater.*, 2020, **32**, 2572-2582
49	1.01	13.96	59	8.32	*J. Mater. Chem. C*, 2020, **8**, 4012-4020
50	1.01	15.66	54.01	8.61	*Angew. Chem. Int. Ed.*, 2020, **59**, 14449–14457
51	0.909	13.19	77.86	9.34	*Nanoscale*, 2020, **12**, 4945-4952
52	1.02	15.16	59.4	9.21	*Sci China Chem*, 2020, **63**, 1785-1792
53	0.967	13.45	70	9.07	*ACS Appl. Mater. Interfaces* 2020, **12**, 16490-16502
54	0.94	17.8	69	11.5	*J. Mater. Chem. C*, 2020, **8**, 16180-16187
55	0.97	17.96	67	12.06	*Adv. Energy Mater.*, 2020, **10**, 2001408
56	0.938	21.5	66.66	13.43	*Sci China Chem*, 2020, **63**, 1449-1460
57	0.85	16.62	67.1	9.46	*ACS Appl. Polym. Mater.*, 2021, **3**, 1923-1931
58	0.85	14.74	69.44	8.73	*J. Mater. Chem. C*, 2021, **9**, 3835-3845
59	0.9	23.3	72.4	15.2	*Angew. Chem. Int. Ed.*, 2021, **60**, 10137-10146
60	0.89	15.83	66	9.31	*Chem. Mater.*, 2021, **33**, 1070-1081
61	0.94	14.1	67	9.02	*Macromolecules*, 2021, **54**, 53-63
62	0.99	15.3	61.4	9.31	*ACS Appl. Mater. Interfaces*, 2021, **13**, 6442-6449
63	1	13.21	67	9.13	*J. Mater. Chem. A*, 2021, **9**, 2775-2783
64	0.87	16.32	65	9.35	*Chem. Eng. J.*, 2022, **428**, 131232
65	0.98	14.56	70.25	10.02	*Sol. RRL*, 2021, **5**, 2100019
66	0.906	20.47	66.3	12.31	*Sci China Chem*, 2021, **64**, 1380-1388
67	0.84	19.21	70.08	11.66	*ACS Appl. Mater. Interfaces*, 2021, **13**, 28231-28241
68	0.92	18.72	63	11.12	*Adv. Energy Mater.*, 2021, **11**, 2003367
69	0.9	21.93	60	11.84	*ACS Appl. Energy Mater.*, 2021, **4**, 4217-4223
70	0.886	21.73	67.62	13.02	*Polymer*, 2021, **230**, 124104
71	0.907	24.82	71.8	16.16	*Nat. Commun.*, 2021, **12**, 5264
72	0.86	22.28	69	13.22	*ACS Energy Lett.*, 2021, **6**, 728-738
73	0.92	22.47	66.7	13.8	*Chem. Commun.*, 2021, **57**, 935-938
74	0.91	22	71.5	14.32	*Chem. Mater.*, 2021, **33**, 761-773
75	0.88	23.27	66.83	14.1	*Adv. Energy Mater.*, 2021, **11**, 2003171
76	0.871	23.6	73.7	15.1	*Angew. Chem. Int. Ed.*, 2021, **60**, 15935-15943
77	0.896	24.7	71.3	15.8	*J. Am. Chem. Soc.* 2021, **143**, 2665-2670
78	0.91	23.2	75	15.8	*J. Mater. Chem. A*, 2021, **9**, 8975-8983
79	0.95	22.1	74.1	15.62	*Energy Environ. Sci.*, 2021, **14**, 4499-4507

80	0.953	22.21	71.86	15.22	*Adv. Mater.*, 2021, **33**, 2102635
81	0.9	22.7	75.3	15.4	*Sci China Chem*, 2021, **64**, 408-412
82	0.88	23.54	73	15.12	*Adv. Energy Mater.*, 2022, **12**, 2103239
83	0.92	22.91	70	14.68	*Adv. Mater.*, 2022, **34**, 2107361
84	0.926	24.1	73	16.3	*Sci China Chem*, 2022, **65**, 309-317
85	0.91	23.07	22.68	16.05	*Nano Energy*, 2022, **93**, 106858
86	0.95	23.82	75.8	17.15	*Energy Environ. Sci.*, 2022, **15**, 3854-3861
87	0.912	24.8	75.8	17.1	*Adv. Mater.*, 2022, **34**, 2205009
88	0.939	24.63	74.96	17.35	*Angew. Chem. Int. Ed.* 2023, e202308306
89	0.94	25.96	76.1	18.56	*Nat. Commun.* 2023, **14**, 10
90	0.938	24.61	79.66	18.39	*Adv. Energy Mater.* 2023, **13**, 2302252
91	0.93	25.95	77.26	18.72	*Adv. Mater.* 2023, 2308334
92	0.934	25.21	76.72	18.06	*Adv. Mater*. 2023, 2307398

Supplementary Table 5. Highest-performing PCE values of solution printed all-PSCs for each year since 2015.

**year**	**J_{sc} (mA/cm2)**	**V_{OC} (V)**	**Fill Factor (%)**	**PCE (%)**	**Reference**
2015	n.d.	n.d.	n.d.	3.2	*Nat. Commun.*, 2015, **6**, 7955
2016	n.d.	n.d.	n.d.	4	*Adv. Energy Mater.*, 2016, **6**, 1601225
2017	0.74	13.77	52.46	5.6	*Adv. Funct. Mater.*, 2017, **27**, 1702016
2018	0.84	14.26	71	8.6	*Nano Energy*, 2018, **46**, 428-435
2019	0.84	14.86	66.65	8.36	*Nano Energy*, 2019, **59**, 277-284
2020	0.883	15.9	61	8.55	*J. Am. Chem. Soc.*, 2020, **142**, 392-406
2021	0.909	22.6	63.4	13.0	*Angew. Chem. Int. Ed.*, 2021, **60**, 10137-10146
2022	0.948	20.21	66.5	12.74	*Sci China Chem*, 2022, **65**, 182-189

Supplementary References

1 F. Neese, F. Wennmohs, U. Becker, C. Riplinger, *J. Chem. Phys.* **2020**, *152*, 224108.

2 A.D. Becke, J. Chem. Phys. 1993, 98, 5648-5652 K. Kim and K. D. Jordan, *J. Phys. Chem.*, 1994, *98*, 10089.

3 P. J. Stephens, F. J. Devlin, C. F. Chabalowski and M. J. Frisch, *J. Phys. Chem.*, 1994, 98, 11623–11627.

4 F. Weigend, R. Ahlrichs, Phys. *Chem. Chem. Phys.*, **2005**, *7*, 3297–3305.

5 S. Grimme, J. Antony, S. Ehrlich, H. Krieg, *J. Chem. Phys.* **2010**, *132*, 154104.

6 S. Grimme, S. Ehrlich, L. Goerigk, *J. Comput. Chem.*, **2011**, *32*, 1456–1465.

7 F. Weigend, R. Ahlrichs, *Phys. Chem. Chem. Phys.*, **2005**, *7*, 3297–3305 .

8 C. M. Breneman, K. B. Wiberg, *J. Comput. Chem.*, **1990**, *11*, 361–373.

9 J. Rivnay, S. C. B. Mannsfeld, C. E. Miller, A. Salleo, M. F. Toney, *Chem. Rev.* **2012**, *112*, 5488.

10 J. L. Baker, L. H. Jimison, S. Mannsfeld, S. Volkman, S. Yin, V. Subramanian, A. Salleo, A. P. Alivisatos, M. F. Toney, *Langmuir* **2010**, *26*, 9146.

11 T. G. Dane, J. Kieffer, & S. Lilliu, (**2020**). *Github*, https://github.com/tgdane/pygix.

12 S. G. Urquhart, A. P. Hitchcock, A. P. Smith, H. W. Ade, W. Lidy, E. G. Rightor and G. E. Mitchell, *J. Electron. Spectrosc. Relat. Phenom.* **1999**, *100*, 119–135.

13 J. Rivnay, S. C. B. Mannsfeld, C. E. Miller, A. Salleo and M. F. Toney, *Chem. Rev.* **2012**, *112*, 5488–5519.

14 E. D. Gomez, K. P. Barteau, H. Wang, M. F. Toney and Y. L. Loo, *Chem. Commun.* **2011**, *47*, 436–438.

15 Cowie, B. C. C.; Tadich, A.; Thomsen, L. The Current Performance of the Wide Range (90-2500eV) Soft X-ray Beamline at the Australian Synchrotron. *AIP Conf. Proc.* **2010**, *1234*, 307–310.

16 B. E. Warren, *X-Ray Diffraction*; Addison-Wesley, **1969**.

17 Watts, B. Calculation of the Kramers-Kronig transform of X-ray spectra by a piecewise Laurent polynomial method *Opt. Express* **2014**, *22*, 23628.

18 Ilavsky, J. Nika: software for two-dimensional data reduction *J. Appl. Cryst.* **2012**, *45*, 324–328.

19 K. Vandewal, K. Tvingstedt, A. Gadisa, O. Inganäs, J. V. Manca, *Nat. Mater.* **2009**, *8*, 904.

20 Dolomanov, O.V., Bourhis, L.J., Gildea, R.J, Howard, J.A.K. & Puschmann, H. (2009), J. Appl. Cryst. 42, 339-341.

21 Sheldrick, G.M. (2008). Acta Cryst. A64, 112-122.

22 Sheldrick, G.M. (2015). Acta Cryst. C71, 3-8.

23 Wang, W.; Wu, Q.; Sun, R.; Guo, J.; Wu, Y.; Shi, M. M.; Yang, W. Y.; Li, H. N.; Min, J. *Joule* **2020**, *4*, 1070–1086.

24 Guo, X. G.; Quinn, J.; Chen, Z. H.; Usta, H.; Zheng, Y.; Xia, Y.; Hennek, J. W.; Ortiz, R. P.; Marks, T. J.; Facchetti, A. *Journal of the American Chemical Society* **2013**, *135*, 1986-1996.

25 Goto, H.; Akagi, K. *Angew. Chem. Int. Ed.* **2005**, *44*, 4322.
