# Supplementary material for: Tuning polymer-backbone coplanarity and conformational order to achieve high-performance printed all-polymer solar cells
Source: Nat Commun. 2024 Mar 9;15:2170. doi: 10.1038/s41467-024-46493-4 (PMC10924936; doi:10.1038/s41467-024-46493-4)
Supplement: Supplementary file 3 — Description of Additional Supplementary Files [file 41467_2024_46493_MOESM3_ESM.pdf]

File name: Supplementary Data 1

Description: Crystallographic Information File (CIF) of model compound Ph-BTz.

File name: Supplementary Data 2

Description: CheckCIF report of model compound Ph-BTz.
