# Supplementary material for: Tuning polymer-backbone coplanarity and conformational order to achieve high-performance printed all-polymer solar cells
Source: Nat Commun. 2024 Mar 9;15:2170. doi: 10.1038/s41467-024-46493-4 (PMC10924936; doi:10.1038/s41467-024-46493-4)

checkCIF/PLATON report

Structure factors have been supplied for datablock(s) mo_yw191_1_abs

THIS REPORT IS FOR GUIDANCE ONLY. IF USED AS PART OF A REVIEW PROCEDURE FOR PUBLICATION, IT SHOULD NOT REPLACE THE EXPERTISE OF AN EXPERIENCED CRYSTALLOGRAPHIC REFEREE.

No syntax errors found. CIF dictionary Interpreting this report

Datablock: mo_yw191_1_abs

Bond precision: C-C = 0.0080 A Wavelength=0.71073

Cell:	a=11.957(3)	b=12.529(4)	c=17.126(6)
	alpha=86.016(7)	beta=84.547(7)	gamma=63.378(9)
Temperature:	100 K		

	Calculated	Reported
Volume	2282.2(12)	2282.3(12)
Space group	P -1	P -1
Hall group	-P 1	-P 1
Moiety formula	C34 H44 N2 O2 S2	0.75(C34 H44 N2 O2 S2)
Sum formula	C34 H44 N2 O2 S2	C25.50 H33 N1.50 O1.50 S1.50
Mr	576.83	432.62
Dx,g cm-3	1.259	1.259
Z	3	4
Mu (mm-1)	0.209	0.209
F000	930.0	930.0
F000'	931.06	
h,k,lmax	17,18,25	17,18,25
Nref	15656	15644
Tmin,Tmax	0.988,0.990	0.645,0.747
Tmin'	0.920	

Correction method= # Reported T Limits: Tmin=0.645 Tmax=0.747
AbsCorr = MULTI-SCAN

Data completeness= 0.999 Theta(max)= 31.850

R(reflections)= 0.0535(4690) wR2(reflections)= 0.1902(15644)

S = 1.014 Npar= 545

The following ALERTS were generated. Each ALERT has the format
test-name_ALERT_alert-type_alert-level.
Click on the hyperlinks for more details of the test.

Alert level B

```
PLAT026_ALERT_3_B Ratio Observed / Unique Reflections (too) Low ..        30% Check
PLAT110_ALERT_2_B ADDSYM Detects Potential Lattice Translation ...          ? Check
PLAT112_ALERT_2_B ADDSYM Detects New (Pseudo) Symm. Elem       b/3        100 %Fit
PLAT113_ALERT_2_B ADDSYM Suggests Possible Pseudo/New  Space Group        P-1 Check
              Check Model Parameter Symmmetry for Reflection Data Support
```

Alert level C

```
ABSTY02_ALERT_1_C  An _exptl_absorpt_correction_type has been given without
            a literature citation. This should be contained in the
            _exptl_absorpt_process_details field.
            Absorption correction given as multi-scan
PLAT230_ALERT_2_C Hirshfeld Test Diff for    C28      --C29      .        5.2 s.u.
PLAT340_ALERT_3_C Low Bond Precision on  C-C Bonds ...............      0.008 Ang.
PLAT905_ALERT_3_C Negative K value in the Analysis of Variance ...     -6.061 Report
PLAT905_ALERT_3_C Negative K value in the Analysis of Variance ...     -0.644 Report
PLAT906_ALERT_3_C Large K Value in the Analysis of Variance ......      3.430 Check
PLAT911_ALERT_3_C Missing FCF Refl Between Thmin & STh/L=    0.600         13 Report
```

Alert level G

```
PLAT045_ALERT_1_G Calculated and Reported Z Differ by a Factor ...      0.750 Check
PLAT116_ALERT_2_G ADDSYM Included (Pseudo) Lattice Translation ...     Please Check
PLAT908_ALERT_2_G Max. Perc. Data with I > 2*s(I) per Res.Shell .      34.60% Note
PLAT910_ALERT_3_G Missing # of FCF Reflection(s) Below Theta(Min).          1 Note
PLAT912_ALERT_4_G Missing # of FCF Reflections Above STh/L=  0.600          1 Note
PLAT913_ALERT_3_G Missing # of Very Strong Reflections in FCF ....          1 Note
PLAT933_ALERT_2_G Number of HKL-OMIT Records in Embedded .res File         10 Note
PLAT967_ALERT_5_G Note: Two-Theta Cutoff Value in Embedded .res ..       63.7 Degree
PLAT978_ALERT_2_G Number C-C Bonds with Positive Residual Density.         15 Info
```

0 **ALERT level A** = Most likely a serious problem - resolve or explain
4 **ALERT level B** = A potentially serious problem, consider carefully
7 **ALERT level C** = Check. Ensure it is not caused by an omission or oversight
9 **ALERT level G** = General information/check it is not something unexpected

2 ALERT type 1 CIF construction/syntax error, inconsistent or missing data
8 ALERT type 2 Indicator that the structure model may be wrong or deficient
8 ALERT type 3 Indicator that the structure quality may be low
1 ALERT type 4 Improvement, methodology, query or suggestion
1 ALERT type 5 Informative message, check

It is advisable to attempt to resolve as many as possible of the alerts in all categories. Often the minor alerts point to easily fixed oversights, errors and omissions in your CIF or refinement strategy, so attention to these fine details can be worthwhile. In order to resolve some of the more serious problems it may be necessary to carry out additional measurements or structure refinements. However, the purpose of your study may justify the reported deviations and the more serious of these should normally be commented upon in the discussion or experimental section of a paper or in the "special_details" fields of the CIF. checkCIF was carefully designed to identify outliers and unusual parameters, but every test has its limitations and alerts that are not important in a particular case may appear. Conversely, the absence of alerts does not guarantee there are no aspects of the results needing attention. It is up to the individual to critically assess their own results and, if necessary, seek expert advice.

Publication of your CIF in IUCr journals

A basic structural check has been run on your CIF. These basic checks will be run on all CIFs submitted for publication in IUCr journals (*Acta Crystallographica*, *Journal of Applied Crystallography*, *Journal of Synchrotron Radiation*); however, if you intend to submit to *Acta Crystallographica Section C* or *E* or *IUCrData*, you should make sure that full publication checks are run on the final version of your CIF prior to submission.

Publication of your CIF in other journals

Please refer to the *Notes for Authors* of the relevant journal for any special instructions relating to CIF submission.

PLATON version of 10/05/2023; check.def file version of 10/05/2023

Datablock mo_yw191_1_abs - ellipsoid plot

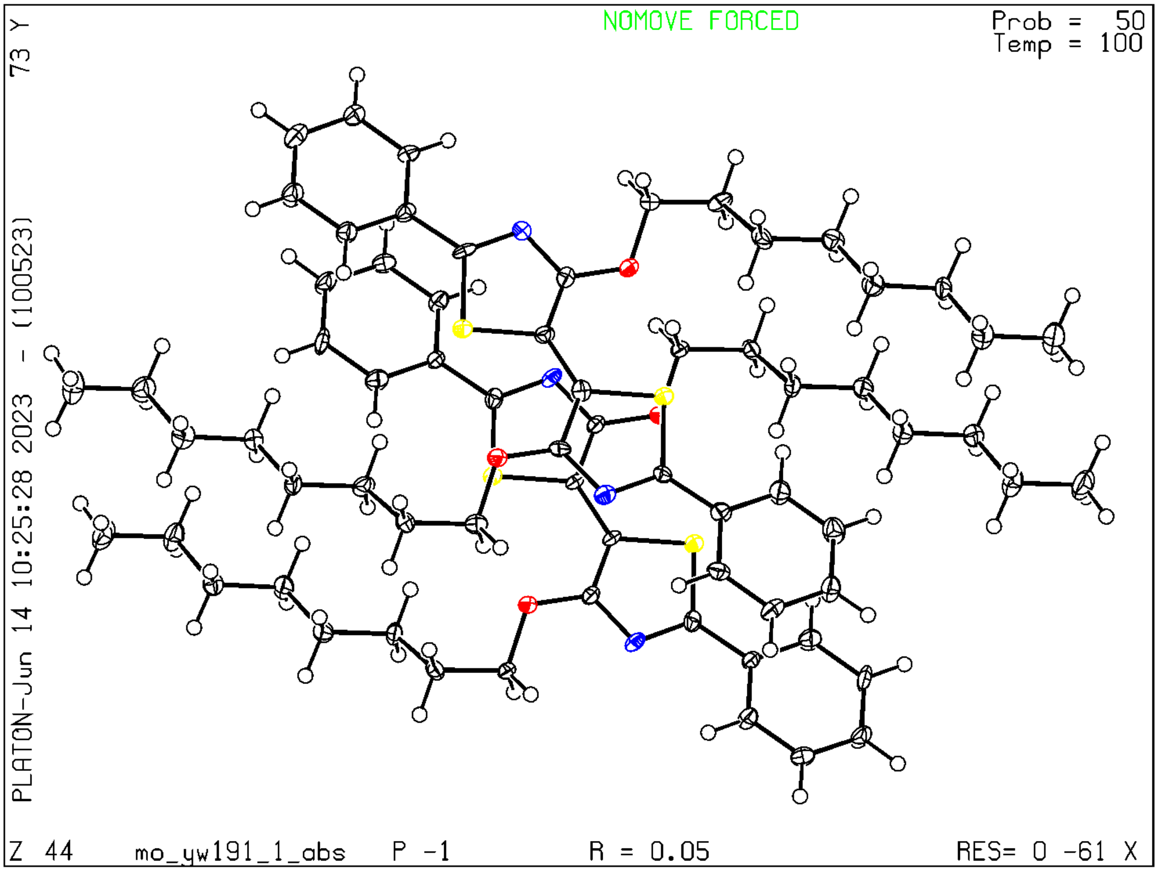

Supplement: Supplementary file 5 — Supplementary Data 2 [file 41467_2024_46493_MOESM5_ESM.pdf]
