# Supplementary material for: Tuning polymer-backbone coplanarity and conformational order to achieve high-performance printed all-polymer solar cells
Source: Nat Commun. 2024 Mar 9;15:2170. doi: 10.1038/s41467-024-46493-4 (PMC10924936; doi:10.1038/s41467-024-46493-4)
Supplement: Supplementary file 6 — Solar Cells Reporting Summary [file 41467_2024_46493_MOESM6_ESM.pdf]

nature research

Corresponding author(s): Zhenan Bao

Solar Cells Reporting Summary

Nature Research wishes to improve the reproducibility of the work that we publish. This form is intended for publication with all accepted papers reporting the characterization of photovoltaic devices and provides structure for consistency and transparency in reporting. Some list items might not apply to an individual manuscript, but all fields must be completed for clarity.

For further information on Nature Research policies, including our data availability policy, see Authors & Referees.

Experimental design

Please check: are the following details reported in the manuscript?

1. Dimensions

Area of the tested solar cells	[x] Yes [ ] No	The device effective area is 4.0 mm2. The description text can be found in the Method Section in the SI.
Method used to determine the device area	[x] Yes [ ] No	Area of the tested solar cells is defined by optical microscope.

2. Current-voltage characterization

Current density-voltage (J-V) plots in both forward and backward direction	[ ] Yes [x] No	Generally, organic photovoltaic devices do not have forward and backward problems. We only scan the device in forward direction.
Voltage scan conditions<br>*For instance: scan direction, speed, dwell times*	[x] Yes [ ] No	The voltage was scanned from -1 V to 1V. The voltage step and delay time were 10 mV and 1 ms, respectively.
Test environment<br>*For instance: characterization temperature, in air or in glove box*	[x] Yes [ ] No	Devices were characterized at room temperature in In N2-filled glove box.
Protocol for preconditioning of the device before its characterization	[ ] Yes [x] No	No preconditioning protocol.
Stability of the J-V characteristic<br>*Verified with time evolution of the maximum power point or with the photocurrent at maximum power point; see ref. 7 for details.*	[ ] Yes [x] No	Organic photovoltaic devices show no decay or instability during the test of J-V characteristics.

3. Hysteresis or any other unusual behaviour

Description of the unusual behaviour observed during the characterization	[ ] Yes [x] No	No hysteresis was observed in our device.
Related experimental data	[ ] Yes [x] No	We didn't find the unusual behaviour.

4. Efficiency

External quantum efficiency (EQE) or incident photons to current efficiency (IPCE)	[x] Yes [ ] No	The data is included in Fig. 3d. We show the EQE measurement details in the Methods section.
A comparison between the integrated response under the standard reference spectrum and the response measure under the simulator	[x] Yes [ ] No	The difference between the integrated current from EQE and the short-circuit current from J-V curve measured under AM 1.5G solar simulator is within 3% difference which is within the accuracy confidence of the measurements.
For tandem solar cells, the bias illumination and bias voltage used for each subcell	[ ] Yes [x] No	We did not make the tandem solar cells in this work.

5. Calibration

Light source and reference cell or sensor used for the characterization	[x] Yes [ ] No	All devices were tested inside a nitrogen glove box under AM 1.5G illumination with an intensity of 100 mW cm−2 (Newport Solar Simulator 94021A) calibrated by a Newport certified silicon photodiode covered with a KG5 filter. Relative information is provided in method section.

Confirmation that the reference cell was calibrated and certified

☒ Yes ☐ No

The reference cell was calibrated and certified.

Calculation of spectral mismatch between the reference cell and the devices under test

☒ Yes ☐ No

By calculating the Jsc from EQE based on the solar simulator spectral for the standard silicon cell and our OSC devices, the mismatch factor was close to unity.

6. Mask/aperture

Size of the mask/aperture used during testing

☐ Yes ☒ No

No, we did not use mask in this measurements. According to previous experience, when we use mask, the device characteristics are equivalent compared with no mask, the variations is within 0.5%.

Variation of the measured short-circuit current density with the mask/aperture area

☐ Yes ☒ No

No, we did not use mask in this measurements.

7. Performance certification

Identity of the independent certification laboratory that confirmed the photovoltaic performance

☐ Yes ☒ No

The photovoltaic performance of our devices was not confirmed from independent certification laboratories.

A copy of any certificate(s)
Provide in Supplementary Information

☐ Yes ☒ No

The photovoltaic performance of our devices was not confirmed from independent certification laboratories.

8. Statistics

Number of solar cells tested

☒ Yes ☐ No

The average PCE of the all-PSCs is obtained from over 10 independent devices.

Statistical analysis of the device performance

☒ Yes ☐ No

We have given statistical data of device performance in the Table 2 and Figure S17.

9. Long-term stability analysis

Type of analysis, bias conditions and environmental conditions
For instance: illumination type, temperature, atmosphere humidity, encapsulation method, preconditioning temperature

☒ Yes ☐ No

The thermal stability of these devices was measured at 90 °C in a nitrogen glove box and included in the Figure S18.
